# Developing a conceptual framework for interdisciplinary communication, collaboration, and integration: A structured approach

**DOI:** 10.1007/s13280-025-02210-z

**Published:** 2025-08-20

**Authors:** Jialin Zhang, Hanna Salomon, Martin Nicola Huber, Harald Bugmann, Julie Elisabet Dölker, Louis König, Jasmin Krähenbühl, Eva Lieberherr, Ivana Logar, Brian McArdell, Peter Molnar, Simone Quatrini, Veronika Schick, Fritz Schlunegger, Chantal Schmidt, Astrid Zabel, Sabine Hoffmann

**Affiliations:** 1 Environmental Social Sciences Department, Swiss Federal Institute of Aquatic Science and Technology (Eawag), Ueberlandstrasse 133, 8600 Dübendorf, Switzerland; 2 Department of Environmental Systems Science, Institute for Environmental Decisions, ETH Zürich, Universitätsstrasse 16, 8092 Zurich, Switzerland; 3 Department of Social Sciences, Institute of Political Science, University of Bern, Schanzeneckstrasse 1, 3001 Bern, Switzerland; 4 Department of Environmental Systems Science, Institute of Terrestrial Ecosystems, ETH Zürich, Universitätstrasse 16, 8092 Zurich, Switzerland; 5 Centre for Development and Environment (CDE), University of Bern, Mittelstrasse 43, 3012 Bern, Switzerland; 6 Mountain Torrents and Mass Movements, Swiss Federal Institute for Forest, Snow and Landscape Research (WSL), Zürcherstrasse 111, 8903 Birmensdorf, Switzerland; 7 Institute of Environmental Engineering, ETH Zürich, Stefano-Franscini-Platz 5, 8093 Zurich, Switzerland; 8 Institute of Geological Sciences, University of Bern, Baltzerstrasse 3, 3012 Bern, Switzerland; 9 Department of Environmental Systems Science, TdLab, ETH Zürich, Universitätstrasse 16, 8092 Zurich, Switzerland

**Keywords:** Boundary concepts, Boundary objects, Collaborative and iterative process, Social-ecological systems, Sustainability transformations

## Abstract

Interdisciplinary research is essential to address the complex environmental challenges faced by social-ecological systems (SES). However, it is often hindered by difficulties in integrating diverse knowledge and perspectives. Conceptual Frameworks (CFs) can act as boundary objects, facilitating integration in contexts with incomplete knowledge, nonlinearity, and divergent interests. Yet,  guidance on developing CFs remains limited. To address this gap, we develop a structured approach and apply it within a research project focused on enhancing the resilience of Swiss Alpine ecosystems. Our approach includes three phases: (1) defining boundary concepts, (2) developing a CF as a boundary object, and (3) using the CF as a boundary object. The resulting CF supports communication, collaboration, and integration across disciplines, advancing SES research that addresses ecological resilience and sustainability. Our approach can be used by other interdisciplinary teams aiming to develop adaptable CFs that facilitate knowledge integration across disciplines.

**Supplementary Information:**

The online version contains supplementary material available at 10.1007/s13280-025-02210-z.

## Introduction

Environmental challenges such as biodiversity loss, climate change, and ecosystem degradation are complex issues caused by multiple interacting factors within Social-Ecological Systems (SES) (Berkes et al. 2008; Virapongse et al. 2016). In this paper, we define SES as integrated complex systems in which people interact with natural components and are an intrinsic part of nature (Liu et al. 2007; Ostrom 2007). The environmental challenges often arise from the dynamic interconnections between social and ecological components, requiring integrative approaches that go beyond single-disciplinary solutions (Costanza and Jorgensen 2002; Hicks et al. 2010; Angelstam et al. 2013). Traditional approaches that attempt to simplify or isolate these components frequently fail because they overlook the full complexity of these interconnected systems (Jessop and Sum 2001; Dick et al. 2016). As the impacts of these challenges become more severe, the need for inter- and transdisciplinary research strategies to address them is growing (Leavy 2016). While interdisciplinary research integrates knowledge and perspectives from multiple academic disciplines (Menken and Keestra 2016), transdisciplinary research extends the integration to include knowledge and perspectives from policy and practice (Hadorn et al. 2008). For this study, we focus on interdisciplinary research.

Despite their advantages, interdisciplinary communication, collaboration, and integration face significant barriers (O’Rourke 2017). Successful interdisciplinary work requires the ability to constructively engage with diverse types of knowledge, perspectives, priorities, and values (O’Rourke et al. 2016). Scientists from different disciplines often employ distinct theories and terminologies, which can result in concepts being interpreted differently (Castellví, 2003; Barnes 2013; Graff 2016). This divergence in interpretation is influenced by perceptual theory orientation, where individuals perceive issues through specific theoretical “frames,” focusing on different dimensions of the same problem, such as technical, operational, or socioeconomic aspects (Bogen 2009). Additionally, semantic theory orientation complicates communication further, as the language used to describe concepts or findings is theory-dependent, with meanings tied to specific theoretical contexts (Bogen 2009). Consequently, scientists with different theoretical backgrounds may use the same terms but attach different meanings to them, significantly hindering effective communication, collaboration and integration. For example, in the case of “water control” (cf., see Mollinga (2010)), a hydrologist or geologist might focus on infrastructure for flood prevention, while a political scientist might interpret it in terms of governance and policymaking. Such divergences emphasize how the same term can be understood differently depending on the researcher’s discipline.

To address the challenges posed by theory orientation in interdisciplinary collaboration, researchers frequently turn to the concept of “boundary work” (Van der Steen and Van Twist 2013, p. 34). Boundary work comprises different elements that facilitate communication, collaboration, and integration across disciplinary boundaries. In this study, we follow the framework of Mollinga (2010), who defines boundary work as being composed of three categories: (1) the development of boundary concepts, (2) the configuration of boundary objects, and (3) the shaping of boundary settings. Boundary concepts are concepts or terms that help researchers think about and communicate complex issues across disciplines (Mollinga 2010). In contrast, boundary objects are practical tools that facilitate action in contexts with incomplete knowledge, nonlinearity, and divergent interests (Mollinga 2010). They are adaptable enough to meet the needs and constraints of various actors (e.g., researchers or stakeholders) while remaining robust enough to maintain a shared identity across different contexts (Star and Griesemer 1989). Lastly, boundary settings are defined as the conditions or institutional arrangements in which researchers from different disciplines can collaborate effectively (Mollinga 2010). One commonly used boundary object in interdisciplinary research is a conceptual framework (CF), which provides a functional and adaptable structure to integrate across disciplinary boundaries (Rossini and Porter 1979; Clark et al. 2016; O’Rourke 2017; Turnhout 2019). CFs enable disciplines to bridge differences effectively by employing adaptable terminology as well as facilitating communication, collaboration, and integration across diverse knowledge systems and perspectives (Pohl and Hadorn 2017). Additionally, CFs support a holistic understanding of complex systems, which is essential for increasing the resilience and adaptability of SESs (Ledesma 2014).

While CFs are widely recognized as valuable for integrating diverse knowledge types and perspectives in interdisciplinary SES research, there is a lack of detailed guidance on how to develop such frameworks that bridge disciplines effectively while serving as boundary objects. Some notable exceptions exist: for example, Bergmann et al. (2012) describe methods for creating a shared understanding through conceptual clarification and building a CF, and Rossini and Porter (1979) and Hoffmann et al. (2017) introduce four procedures to integrate knowledge in interdisciplinary research. These studies underscore that collaborative and often iterative processes can facilitate interdisciplinary communication, collaboration and integration. Hertz and Schlüter (2015) even propose a two-step approach to test the suitability of the SES framework as a boundary object. However, explicit, practical guidance for interdisciplinary teams on how to develop a CF in a collaborative and iterative process remains scarce.

To address this gap, we present a structured approach to develop CFs specifically designed to bridge disciplinary boundaries in SES research. Our approach is deliberately iterative and collaborative, reflecting the notion that integrating diverse knowledge types and perspectives requires repeated cycles of joint reflection and synthesis (Raymond et al. 2010; Tengo et al. 2014). By detailing our approach as a multi-step iterative and collaborative process, in which different methods and procedures are constructively combined (Hoffmann et al. 2017), we aim to advance the use of CFs as boundary objects to enhance communication, foster collaboration, and support meaningful knowledge integration. Below, we first detail our approach for developing a CF, highlighting each step in the process. The term “process” here refers broadly to our iterative and collaborative efforts to develop, refine, and apply the CF throughout the project. Then we demonstrate the application of the CF through a case study focused on a specific environmental challenge, highlighting its effectiveness as a boundary object to foster communication, collaboration, and integration among diverse researchers from different disciplines. Finally, we discuss the broader implications of our structured approach and explore potential future applications across diverse environmental and societal contexts. By doing so, we aim to contribute to the broader field of interdisciplinary research methodologies, offering a structured approach for researchers navigating the complexities of knowledge integration across diverse disciplines.

## Materials and methods

### Case study

We use the TREBRIDGE project, formally titled *Transformation toward Resilient Ecosystems: Bridging Natural and Social Sciences*, as a case study. Aiming to enhance resilience in Swiss Alpine SES, the project offers a real-world laboratory (Schäpke et al. 2018) to apply and refine our approach to developing an interdisciplinary CF. The project’s primary objective is to *“develop pathways for rethinking future management, aiming for higher resilience of Alpine ecosystems and delivering greater societal value than current systems.”* (Lieberherr et al. 2025, p. 35).

This objective has been derived from the problem statement that *“[f]or centuries, humans have engineered Alpine watersheds for erosion and flood control by building check dams in streams and replanting clear-cuts with monocultures. As a result, large areas are covered by single-species, even-aged coniferous forests, channelized streams controlled by dams and artificial banks, and numerous roads to access dam sites and forest resources. These landscapes require large investments to maintain check dam infrastructure and forest operations.”* (Lieberherr et al. 2025, p. 35). At the same time “*[t]his infrastructure leads to an unnatural state of these watersheds, raising concerns about ecosystem quality and resilience”* (Lieberherr et al. 2021).

The project is divided up into five work packages (WPs). WP1 is responsible for science integration as part of the project’s inter- and transdisciplinary approach—also referred to as integration leaders in this paper. WP2 focuses on participatory scenario development, including stakeholders from outside academia; WP3 investigates sediment production and transfer in Alpine watersheds; WP4 explores the hydrological and ecological consequences of Alpine watershed management; WP5 investigates environmental policy and management options, along with the values of nature and justice aspects, while incorporating input from stakeholders beyond academia. The project brings together researchers from geomorphology, forest ecology, hydrology, environmental economics, natural resource policy, and inter- and transdisciplinary research (Lieberherr et al. 2025). Given the complexity of integrating knowledge across these disciplines, TREBRIDGE requires a shared framework that accommodates diverse disciplinary perspectives. The CF serves as a boundary object that bridges differences across and even within disciplines, facilitating effective interdisciplinary integration. To develop the CF, a structured yet flexible approach to foster communication, collaboration, and integration is needed.

### Developing the conceptual framework

The CF development approach in TREBRIDGE is structured to foster interdisciplinary communication, collaboration, and integration through a collaborative and iterative process. It is informed by established theories on interdisciplinary integration, particularly the integration procedures (originally termed “socio-cognitive frameworks”) as proposed by Rossini and Porter (1979) and Hoffmann et al. (2017), as well as the integration methods outlined by Bergmann et al. (2012). Below, we summarize each of these foundational theories, which form the basis of our approach.

Based on Hoffmann et al. (2017), Hoffmann (2024, p. 507), we outline three procedures for integrating knowledge in interdisciplinary research:

**Table:** 

	*Common group learning, i.e.* insights provided by researchers from different disciplines are integrated and synthesized within the group as a whole; the final product represents the common integrated knowledge of the entire group
	*Negotiation among experts, i.e.* insights are combined through bilateral interactions among researchers at the boundaries between their individual expertise; the final product reflects the researchers’ disciplinary background with informed and interrelated analyses at the boundaries of the researchers’ respective expertise
	*Integration by a leader, i.e.* insights are integrated and synthesized by a leader (or a small team of co-leaders) through bilateral interactions between the leader and each researcher, but without researchers’ interactions among themselves; the final product represents the integrated knowledge of the leader (or the small team of co-leaders)

These procedures provide a framework for organizing effective knowledge integration across diverse disciplines within a team.

Bergmann et al. (2012) describe two key integration methods that promote interdisciplinary understanding through conceptual clarifications and theoretical frameworks:

- *Interdisciplinary and discipline-specific clarification of important concepts and terms*, which is drawn from the problem field: this involves identifying and defining key terminologies and ideas central to the research problem, considering both interdisciplinary perspectives and discipline-specific nuances.
- *Integrative theoretical framework*, which starts by de-contextualizing discipline-specific concepts, analyzing and generalizing them for broader use, followed by re-contextualizing them to ensure that they remain relevant within each discipline.

This emphasis on iterative concept clarification and framework building in prior studies informs the design of our approach. In line with general observations that interdisciplinary framework development proceeds through iterative cycles of concept identification and refinement, repeated rounds of team input and adjustment are at the core of our approach.

**Fig. 1:**
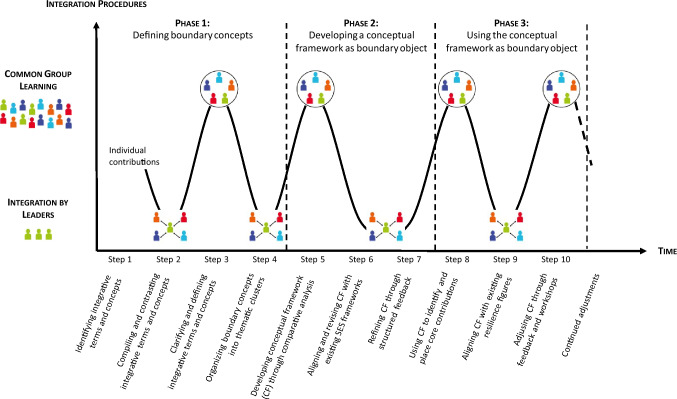
Visualization of the integration procedures of TREBRIDGE researchers and integration leaders during the different steps of the process of developing a conceptual framework. If only integration leaders (green silhouettes) are involved in a step, knowledge and perspectives get integrated in the setting of “integration by leaders.” If all TREBRIDGE researchers are involved, this takes place in the setting of “common group learning.” Red, orange, and green silhouettes represent social scientists, light and dark blue silhouettes represent natural scientists. Figure inspired by Krütli et al. (2010) with elements from Hoffmann et al. (2017)

The approach is structured to bridge disciplinary divides and applied in an iterative and collaborative process consisting of ten detailed steps (see Fig. 1). Rather than creating the framework in one go, the team proceeds in cycles through such steps, including feedback loops, progressively refining both the boundary concepts and the CF as boundary object itself. The iterative nature of the process, alternating between “integration by leaders” and “common group learning” (Hoffmann et al. 2017; Hoffmann 2024), enables the CF to evolve with ongoing team input, ensuring its relevance and adaptability to the project’s dynamic needs. It is important to note that “negotiation among experts” is deliberately not a formal part of this process. Instead, the approach prioritized iterative refinement of both boundary concepts and boundary object within the group, avoiding potential conflicts or power dynamics that might arise from direct negotiation—such as senior researchers dominating discussions, or disciplinary hierarchies undervaluing certain knowledge.

Below we outline the three phases of boundary work to develop our CF: (1) defining boundary concepts, categorized as “knowledge for understanding” (Mollinga 2010), (2) developing a CF as a boundary object, categorized as “knowledge for doing” (Mollinga 2010), and (3) using the CF as a boundary object throughout the research project.

### Phase 1: Defining boundary concepts

Phase 1 primarily addresses the challenge of semantic differences in integrating interdisciplinary knowledge. They arise due to the theory-laden nature of language and concepts, where different disciplines attribute varying meanings to the same terms. For example, “when someone does not know the meaning of another community’s terms or when someone is not aware of how a word changes meaning depending on the context.” (Pohl and Hadorn 2008, p. 115). Nurius and Kemp (2019, p. 173) attribute this to the “narrowing effect of specialized training”. This challenge is also perceived to be present in TREBRIDGE, as mentioned by a TREBRIDGE researcher (Salomon 2023):

*“I think it’s really a challenge, as trivial as it sounds, to understand each other. […] Everybody comes with his/her own perspective, and that’s the way it should be, but to first recognize, okay, that’s actually a different perspective, to break it down, to understand it, and then to create the connections between the different perspectives, that’s a challenge, I think.”*

To address these challenges, we have identified boundary concepts and have compiled them in a glossary, which aligns with Bergmann et al. (2012, pp. 62–63)’s integration “*method of interdisciplinary and discipline-specific clarification of important terms and concepts*”. We regard this method as crucial to finding common ground and fostering understanding of the multidimensional nature of sustainable transformation toward resilient ecosystems, as highlighted by team members, for example, as follows: *“many of the concepts are very different, depending on who makes use of them or describes them”*.

#### Step 1: Identifying integrative terms and concepts

Initial identification of some integrative terms and concepts such as “resilience” had already occurred during the proposal writing and revision process, which some senior researchers were involved in. After the project start, experts in inter- and transdisciplinary research (here called “integration leaders”) asked each team member to identify and define (a) disciplinary concepts (such as “policy instruments” or “debris flow”) that are central to their discipline and should be understood by the other disciplines to enable cross-disciplinary exchange, and (b) integrative terms and concepts (such as “resilience” or “transformation”) that are central for the envisioned integration and synthesis within the project and for which the team was to develop a joint definition. The integration leaders collected these terms and concepts defined in accordance with their respective disciplines.

#### Step 2: Compiling and contrasting integrative terms and concepts

The integration leaders compared the definitions of integrative terms and concepts provided by the team members, merging overlapping ones, keeping contradictory ones, and identifying open questions to be discussed in a common group learning setting (step 3). One example of a somewhat contradictory term that the integration leaders identified was “transformation” (see Appendix A for all definitions). Two team members (one from natural sciences, one from social sciences) sent in very short definitions such as “The change of a system from one state (where we are) to another state that is more desirable (where we want to be)”, based on Pohl (2022) and “Large-scale societal change, often involving a fundamental shift in the interaction between humans and their environment,” based on Hölscher et al. (2018). Two other social scientists expanded on the definition of “transformation” by including different dimensions (normative, analytical, strategic) and describing the process of transformation more in-depth, for example, as follows: “Transformation of a system refers to a process altering fundamental aspects of a system, such as its dynamics or function. This transformation can occur due to endo and /or exogenous changes, i.e., changes within the system itself or independent external variable changes”, based on Beisner et al. (2003). Open questions that were formulated by the integration leaders in the case of the concept of “transformation” included, for example: “Do you agree with the three dimensions (normative, analytical, strategic) of the transformation concept? What do these three dimensions mean for TREBRIDGE? Which dimensions should we consider?” (see Appendix A).

#### Step 3: Clarifying and defining integrative terms and concepts

Based on the compiled table of integrative terms and concepts, the entire team engaged in discussions to address the open questions, refine concepts, unify definitions and in some cases reach consensus. This was achieved during an integration workshop that took place in December 2022, roughly three months after the project had started. The TREBRIDGE research team split into two sub-groups and discussed diverging definitions, open questions, or issues of a subset of the compiled integrative terms and concepts, such as “ecosystem”, “transformation”, and “risk”. This process led to some definitions being adapted and changed and others being moved from the integrative to the disciplinary concepts (e.g., “economic values of nature”) as they did not spark any discussions among the team members and were central to only one discipline involved in the project (e.g., “environmental economics”). A few new concepts previously labeled as disciplinary were added to the integrative part of the glossary (e.g., “benefits” and “nature-based solutions”) during the discussions as they were central to different disciplines and key for the envisioned project synthesis.

At the end of the workshop, the integration leaders of the two sub-groups summarized in the plenum the changes made to the definition of integrative terms and concepts compiled in the glossary (for an example for the term “transformation”, see Appendix A) and emphasized that the glossary is a working document and thus not static.

#### Step 4: Organizing boundary concepts into thematic clusters

In this step, the integration leaders reviewed the glossary from step 3 to identify boundary concepts critical for understanding SES dynamics within Alpine ecosystems relevant to TREBRIDGE. The integration leaders then organized these into thematic clusters. They prioritized core boundary concepts such as “resilience”, “transformation”, “risk”, and “action-oriented knowledge” for clustering based on their relevance to the project’s objectives. They then grouped these concepts into six clusters: (1) Socio-Ecological Systems (SES); (2) Nature’s Contributions to People (NCPs); (3) Action-Oriented Knowledge; (4) Transformation and Resilience; (5) Scenarios and Drivers of Change; (6) Vulnerability, Risk, Benefits, and Values (see Appendix B for a detailed explanation of each cluster).

Each cluster provides a thematic lens essential for understanding SES in the context of TREBRIDGE. For example, the SES cluster focuses on the foundational interactions between social and ecological systems, while the NCP cluster examines NCPs that directly impact human well-being. In addition, the clusters also act as integrative units, facilitating the systematic incorporation of insights from different disciplines into the conceptual framework. For instance, “transformation”, “resilience”, “scenarios”, and “drivers of change” represent interdisciplinary themes that support the analysis of system dynamics and long-term planning. In practice, each cluster served as a building block for subsequent CF development. Team members could see how these themes overlap or remain distinct across disciplines, triggering reflections on potential synergies between disciplines.

Next, the integration leaders presented the clusters and the related definitions to the team members from each WP. This step allowed team members to suggest adjustments, ensuring that the framework captured the research problem accurately from multiple disciplinary perspectives. It completed the initial phase of identifying boundary concepts, thus setting the foundation for collaboratively developing a conceptual framework.

### Phase 2: Developing a conceptual framework as a boundary object

While phase 1 focused on addressing semantic differences through the development of boundary concepts, the next challenge was to create a shared structural foundation that integrates these diverse boundary concepts into a cohesive framework. In interdisciplinary projects, perceptual differences often arise as team members interpret concepts and issues from their unique disciplinary and personal perspectives, leading to varied understandings of key components and interactions within the project. These differing perspectives can fragment team efforts and inhibit a comprehensive understanding of the research objectives. As one of the team members said: “*We each see the project through our [own] lens—what looks like an ecological problem to one of us might seem like a social or economic issue to someone else*.” To bridge these perceptual divides, phase 2 centered on developing a CF that functions as a boundary object.

#### Step 5: Developing a conceptual framework through comparative analysis

In this step, integration leaders utilized the thematic clusters from step 4 as blocks to construct a rough CF which was refined and tested through a participatory method (cf. Pennington et al. (2021)), where each WP team compared perspectives to improve the framework’s design. The objective was to ensure that the framework effectively addressed the research questions in TREBRIDGE while also incorporating discipline-specific questions and definitions identified in the previous steps. To achieve this, semi-structured interviews (see Appendix C) were conducted with members of the different WPs within TREBRIDGE between May and June in 2023.

**Fig. 2:**
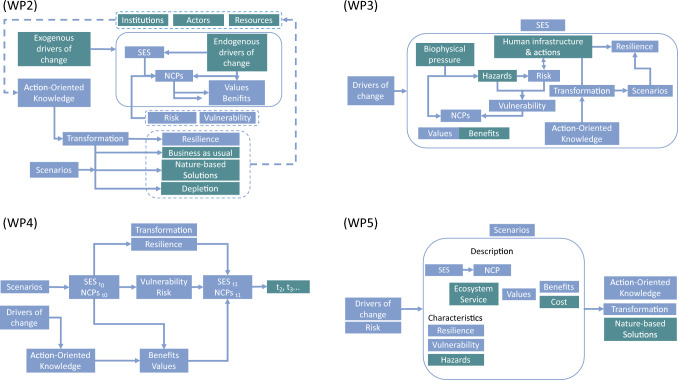
Flowcharts illustrating alternative proposals for the TREBRIDGE conceptual framework (CF) developed by the work packages (WPs) through semi-structured interviews. The blue boxes indicate boundary concepts identified in step 3, while the green boxes represent new terms/concepts added by WPs. WP2: Natural Resource Policy. WP3: Geology and Geomorphology. WP4: Plant Ecology & Modeling. WP5: Environmental Economics. NCP: Nature’s Contributions to People; SES: Social-Ecological Systems

First, the integration leaders encouraged each WP team to collaboratively develop their own CF. The integration leaders instructed the team members to use the six clusters from step 4 and identify connections between the boundary concepts within each cluster, as well as interactions between the clusters. These connections and interactions were determined based on their understanding of the research problem and the specific research questions they aim to address. Drawing such connections and interactions enabled the team members to bring in an unfiltered view of their diverse understanding of the research problem, fostering the exploration of various disciplinary perspectives and the emergence of novel insights (Fig. 2).

Finally, the integration leaders, along with the team members, examined the similarities and differences among these initial CFs during semi-structured interviews. This comparative analysis allowed team members to assess whether their research questions in the TREBRIDGE project were adequately considered when developing the framework and whether the framework accurately captured the diverse understandings of the research problem to be addressed. The insights and feedback collected through this process led to the first version of the CF (hereafter referred to as CF_i, see Appendix D for the different versions of the CF), integrating the unique perspectives and interdisciplinary connections identified by each WP.

#### Step 6: Aligning and revising the conceptual framework with existing SES frameworks

In this step, the integration leaders reviewed the literature on SES frameworks to ensure that the CF aligned with existing theories and practices, such as Ostrom’s SES model (Ostrom 2009) and the resilience theory by Folke et al. (2016). Core concepts related to resilience, human-nature interactions, and transformation were extracted and systematically mapped to the CF’s initial structure. This process clarified how SES principles could be integrated, particularly focusing on the interplay between social and ecological subsystems.

Using both team feedback from the previous steps and the literature-based insights in this step, the integration leaders refined the CF to create a second version with a clear visualization that served as a boundary object (hereafter referred to as CF_ii). The integration leaders presented this updated version in a project workshop in June 2023 to collect detailed feedback from all team members, which helped the integration leaders to refine the CF by the integration leaders. To conclude this step, the integration leaders designed an online survey (see Appendix E) and distributed it to team members. Its primary focus was to assess how project members perceived the framework’s value as a boundary object, acting as a practical tool to enhance communication, collaboration, and integration. In addition to evaluating its usefulness, the survey also aimed to capture specific challenges, benefits, and opportunities experienced during its development. The result of the survey is presented in the “Recommendations for developing future conceptual frameworks” section.

#### Step 7: Refining the conceptual framework through structured feedback

The integration leaders reviewed the feedback gathered from the workshop sessions in step 6. It addressed various aspects, including the practical applicability of concepts and the relevance of connections within the CF. Based on this feedback, the integration leaders adjusted specific components of the CF to better incorporate all disciplinary perspectives. Key modifications included refining visual representations of SES interactions and further classifying the NCPs to reflect both positive and negative contributions (hereafter referred to as CF_iii, see Appendix D).

After making these adjustments, the entire project team summarized the CF development and submitted a manuscript for peer review in an international journal. The subsequent peer review feedback prompted a re-evaluation of the CF’s purpose and broader value beyond TREBRIDGE’s immediate goals. In response, the integration leaders shifted focus to highlight the CF development process as a key contribution to interdisciplinary scholarship. Recognizing the limitations of a “one-size-fits-all” framework for complex SES contexts, they emphasize the iterative, collaborative, and integrative nature of identifying boundary concepts and developing the CF as a boundary object. This process orientation underscores the CF’s role in facilitating ongoing learning and decision-making, offering a structured, flexible approach for future interdisciplinary projects (Mollinga 2010).

### Phase 3: Using the conceptual framework as a boundary object

With the initial CF established in phase 2, the focus of phase 3 shifted to applying it as an adaptable boundary object to support further integration throughout TREBRIDGE. While a static framework may provide initial orientation, its effectiveness may diminish if it fails to adapt to the evolving insights and complexities that emerge during interdisciplinary work. By using the CF as a “living device,” the TREBRIDGE team aims to ensure that it remains responsive to new data, disciplinary insights, and contextual changes, thereby supporting ongoing interdisciplinary learning and decision-making.

This phase has encouraged a collaborative process in which the CF remains relevant and actionable. As one TREBRIDGE researcher reflected, “*Seeing how the framework changes [as we discuss it] has been valuable. It’s not just a finished product—it’s a tool we’re shaping together, and that’s important for our project.*”

#### Step 8: Using the conceptual framework to identify and place core contributions

The CF from phase 2 (CF_iii, see Appendix D) was used during a retreat with the entire TREBRIDGE team. As preparation for the synthesis session, an integration leader identified—based on the initial research proposal—each WP’s expected core contributions to address the research problem at stake, discussed these contributions bilaterally with each WP team and synthesized them to four to six core contributions for each WP. Each WP was given the preparatory exercise to place their core contributions in the CF from step 7. They were provided with the CF (CF_iii, see Appendix D), a short summary of it as well as the glossary definitions of integrative terms and concepts in the CF.

During the session itself, each WP placed sticky dots in the CF, which had been printed on a large poster. While placing them, they explained their reasoning; sometimes a short discussion emerged when other WPs would place a certain core contribution differently. After all WPs had placed their core contributions, a plenary discussion arose about how the framework should be adjusted to better fit TREBRIDGE (see “Conceptual framework” section for a detailed description).

#### Step 9: Aligning the conceptual framework with existing resilience figures

Following the synthesis session, an integration leader revised the CF from step 8 (CF_iv, see Appendix D) based on the feedback and the proposed changes using a recording of the retreat session and the CF poster with suggested changes from the previous step leading to CF_v (see Appendix D). This revised version of the CF was then circulated among the integration leaders. During this review, the integration leaders incorporated additional changes, for example, informed by the literature recommended by team members related to existing visualizations of SES resilience. This iterative process led to a refined version of the CF (CF_vi, see Appendix D), which served as a foundation for broader team engagement.

#### Step 10: Adjusting the conceptual framework through feedback and workshops

**Fig. 3:**
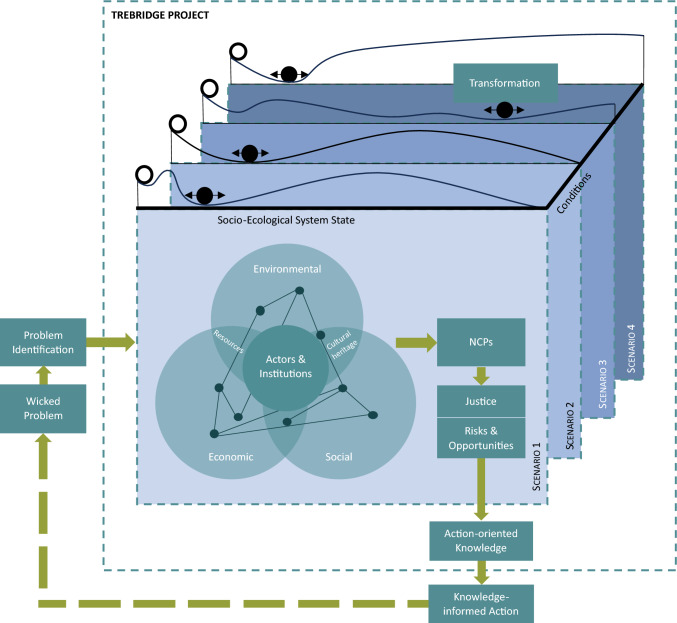
The conceptual framework (CF) as it is used as boundary object in the TREBRIDGE project after multiple initial development process steps. It starts with a Wicked Problem, one specific problem of which is identified (left side of figure). The CF connects Actors and Institutions across the Environmental, Economic and Social dimensions culminating in SES (circles on the left side), which have a certain resilience landscape (wavy lines on top of each Scenario layer). Resilience landscapes are inspired by Scheffer et al. (2001). These landscapes are described by the Socio-Ecological System State and Conditions such as Context Factors and Drivers of Change. In TREBRIDGE, four Scenarios with the same starting point (transparent ball) are analyzed through the lens of Nature’s Contribution to People (NCPs) and the aspects of Justice as well as Risks & Opportunities (boxes on the left of the circles), resulting in different inferred resilience states for the scenarios depicted by the solid black balls. These get attracted to the valleys in the resilience landscape, according to the principle of gravity (Dölker 2022). Changes in the system state would result in a Transformation (double-sided black arrow). This analysis leads to Action-oriented Knowledge that can inform stakeholders and spark Knowledge-informed Action, which in turn influences the Wicked Problem. The time component of the research process starts with the Problem Identification and goes clockwise along the green arrows. The time component of the SES is shown with the transparent balls all starting from the same initial SES State and the bi-directional black arrows depicting Transformation. The latter is therefore not unidirectional and would go hand in hand with a change in time

In this final phase, the CF was iterated several times in quick succession with input from TREBRIDGE junior researchers and integration leaders, resulting in a new visualization (CF_vii, see Appendix D). This version was then discussed with the whole TREBRIDGE research team in a common group learning setting during a webinar in November 2024. Aspects such as the resilience concept were discussed intensively again, ending with agreement on a visualization analogous to the one by Scheffer et al. (2001). It was mentioned that the lack of the time dimension in this visualization was difficult to grasp for some team members and led to misreading the visualization. To tackle this issue, the integration leaders decided that a written explanation of the conceptual framework could be helpful to understand and grasp the concept and the CF better. The integration leaders developed such a summary after the webinar (see caption of Fig. 3). The collaboratively refined CF (CF_viii), now cohesively visualized (see Fig. 3), provides a unified structure for the ongoing interdisciplinary work in the project while still being adaptable to future changes that may occur during the project work.

## Results

### Conceptual framework

The CF (CF_viii) that resulted from all three phases of boundary work (see Fig. 3) can be read as follows: the TREBRIDGE project conceptualizes Alpine ecosystem management as a “wicked problem”, incorporating multiple interacting factors and uncertainties (see “Wicked Problem” and “Problem Identification” boxes on the left side of Fig. 3). The TREBRIDGE research team examines environmental, social, and economic context factors and drivers of change in relation to actors and institutions (see circles in Fig. 3). Based on these context factors and drivers of change, we have developed a range of scenarios on what future Alpine ecosystems could look like (see layers in Fig. 3). Each scenario will result in a corresponding resilience landscape that reflects how an SES responds to specific context factors and drivers of change. One example of these context factors are environmental factors, such as the increased frequency of extreme weather events like heavy rainfall, which impact soil stability and disrupt ecosystem services. These resilience landscapes are not static; they evolve over time as the system adapts or transforms under different conditions. Depending on the scenario, a given landscape may transform dynamically through time, shaped by the drivers of change and their interactions with context factors. Alternatively, a landscape may remain relatively stable if context factors and drivers of change are managed or adapted accordingly. The dimension of time is critical in this framework, as it underscores the evolving nature of these landscapes and their associated SES states. For each scenario, we assess values for different NCPs and analyze aspects of justice as well as risks and opportunities of Alpine SES (Fig. 3, boxes on the right of the circles). These assessments inform the positioning of the black balls representing the current state of the SES within their respective resilience landscapes, which represent the inferred resilience of each scenario (the scenario layers in Fig. 3). Combining this knowledge with insights into the environmental, social, and economic systems, we seek to provide action-oriented knowledge. This knowledge informs stakeholders about how they could use it for knowledge-informed actions (boxes in the bottom-right corner in Fig. 3). The implementation of such actions feeds back to the nature of the wicked problem and may create new “problems” that can be researched in a similar manner (dashed arrow in Fig. 3).

**Fig. 4:**
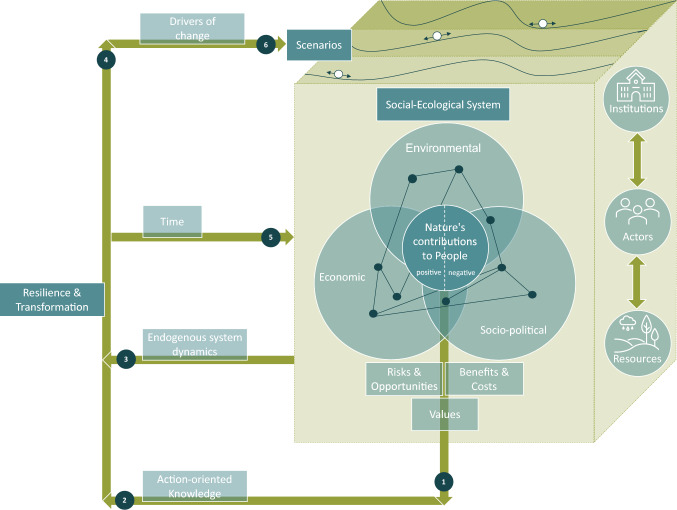
The conceptual framework (CF) as it was used as boundary object in the TREBRIDGE project during a retreat (see step 8)

The CF has evolved considerably throughout the iterative and collaborative process. Overall, we observed a trend toward a simplification of the CF during common group learning procedures, whereas the CF got more complex during the integration procedures carried out by integration leaders. As documenting changes from all iterative steps goes beyond the scope of this paper, changes from one step (step 8) are described here as one example of this collaborative process. This step was chosen for detailed documentation because it marked the first time the integration leaders presented a draft version of the CF (see Fig. 4 or CF_iii in Appendix D), which was then discussed with the TREBRIDGE team in a common group learning procedure. During the discussion, the overall structure of having institutions, actors, and resources separate on one side of the cube was questioned. This was reconciled quickly as everyone agreed to move actors and institutions to the middle of the three dimensions (environmental, economic, and new: social instead of socio-political dimension), and resources to overlap with the environmental and economic dimensions. The NCPs that were initially in the middle of the CF presented were placed so that they emerged from the interplay of the three dimensions and actors and institutions in the middle.

The team members also questioned the explicit placing of the term endogenous system dynamics, as it is inherently contained in the resilience landscapes of the different scenarios on the top of the cube. Therefore, they agreed to omit the term in the visualization. Interestingly, this discussion made underlying assumptions about time scales explicit. For example, different WPs look at different time scales—from seasonal changes to changes over decades and even thousands of years. However, there was also a discussion about the term resilience and its visualization that was not conclusive. Different examples and approaches were shown that were then used by the integration leaders in a next step to make modifications to the resilience landscapes (see step 9).

### Added value of the conceptual framework

The CF provides an added value for the TREBRIDGE project in multiple ways. First, the CF provides a procedural added value, as it supports the ongoing research in an integrative manner. When team members were asked during the retreat session (in August 2024) how the CF resulting from step 7 (CF_iii in Appendix D) had influenced their work so far (step 8), several mentioned that it has had a positive influence, ranging from sparking discussions and therefore supporting communication to influencing one researcher’s way of teaching based on a more holistic view:

*“During excursions in the field of natural hazards, I try to include the human aspect (how to deal with natural hazards, inclusion of environmental aspects, spatial planning, political preferences, stakeholders, costs, etc.). Various actors at different levels are involved, which makes discussing such issues more difficult. The Conceptual Framework provides a framework for organizing these aspects and establishing causal relationships between them.” (written statement by a TREBRIDGE researcher when followed up on oral statement during retreat session (step 10))*

Another TREBRIDGE researcher stated during the retreat session that:

*“The CF helps even though you feel your research might not be 100% covered, [...] This is the experience I have from other projects; it helps to see the bigger picture. Because you see even though I’m contributing here [pointing to contribution in environmental sphere], I need to be aware of the economic and socio-political. So, I need to think in an interconnected manner.”*

Further team members see added value in the CF because *“it’s influencing the way we communicate and we interact [and] we understand each other”*, for example by bringing different perceptions and ideas to light and starting discussions about important aspects such as the resilience of SES in different scenarios. Further, according to one team member, it enhanced the understanding of what others work on exactly.

Second, from the integration leaders’ perspectives, the structured approach and iterative and collaborative process lead to the integration of divergent perspectives on key concepts (e.g., resilience, transformation), achieving consensus where possible and clarifying differences where necessary (cf. step 3). The glossary has served as an essential tool, fostering mutual understanding and supporting the framework’s adaptability.

Going forward in TREBRIDGE, we plan to use the CF as a guiding framework when integrating different knowledge types (system, target, and transformation) (ProClim 1997) and producing integrated outputs. However, the application of the framework throughout project implementation presents a significant challenge. Often, interdisciplinary CFs are used selectively, primarily to provide a lens for thinking about the research topic. However, to fully leverage the potential of a CF, it is crucial to extend its application across the entire project, ranging from defining complex problems to generating, assessing, disseminating, and using new knowledge (Hoffmann et al. 2019). This holistic application requires a persistent commitment to critical reflection and continuous adaptation of the CF during the entire project. This could still have been improved in TREBRIDGE, as one researcher stated that s/he had *“the feeling that we just have to maybe bring it to life a little more. I have the feeling that [so far] we will only get to it if you [integration leaders] [actively] involve us.”* It is important to note that the application of this CF represents an open-ended process: the CF is designed to be adaptable, recognizing that inter- and transdisciplinary research is a constantly evolving endeavor.

## Discussion

Below, we compare our approach and the resulting CF (CF_viii, see Fig. 3) with the literature to identify areas of alignment and divergence from previous approaches and processes. Finally, we briefly explore the broader impact of this structured approach on interdisciplinary research practices and CF development and address its limitations.

### Alignment and divergence from existing processes

A first key alignment between our approach and existing interdisciplinary literature is the recognized importance of developing a shared vocabulary to overcome disciplinary terminology differences (Bergmann et al. 2012). Each scientific discipline has its unique terminology, which optimizes communication within the discipline but can pose barriers to researchers from other domains. As scientific disciplines evolve, their terminology becomes increasingly specialized (Plavén-Sigray et al. 2017; Tobi and Kampen 2018), further exacerbating this challenge. In TREBRIDGE, defining boundary concepts and compiling them into a glossary (phase 1) directly addressed this issue. Unlike static glossaries compiled post-hoc, our glossary is continuously refined based on ongoing team feedback, significantly reducing misunderstandings and facilitating clearer dialog. Our approach aligns with Pohl and Hadorn’s (2008) recommendation to maintain living glossaries or shared terminology lists that are updated throughout a project. It also echoes the study by Freeth and Caniglia (2020), who call for adaptive methodologies in transdisciplinary research to accommodate evolving insights and allow for terminological adjustments. In summary, the emphasis we placed on co-creating and iteratively updating a shared language mirrored best practice and demonstrated how a structured approach to clarifying terminology can significantly enhance interdisciplinary communication.

A second alignment with the literature is the use of an adaptable boundary object through an iterative and collaborative process. Indeed**,** central to our approach was the iterative and collaborative process, consistently implemented from defining boundary concepts (phase 1), through the development of the CF (phase 2), and its ongoing application and refinement (phase 3). This aligns with existing research emphasizing that integrating diverse types of knowledge inherently requires repeated cycles of joint reflection and synthesis (Raymond et al. 2010; Tengo et al. 2014). This iterative approach ensures that the CF remains adaptable, which it should be as a boundary object. More importantly, our iterative process highlights the adaptability of boundary objects over time. A notable example is how we introduced the concept of “justice” into the framework in later iterations as the team recognized its importance—an addition that was not part of the initial framework. This ability to integrate new aspects (like justice) as the project evolved is a key strength of the iterative process used to develop the TREBRIDGE CF. It demonstrates how a boundary object can grow and change while still serving as a ‘practical tool’ to facilitate communication, collaboration, and integration. This finding extends previous work by showing that with a structured yet flexible approach, a CF can remain dynamic and responsive to emerging topics without losing its integrative function. To operationalize this theoretical insight into practical application, we implemented a series of actions, including multiple workshops, group interviews, and a survey (steps 4–10). This explicit operationalization clarified that our iterative process—and thus the adaptability of the CF—was not merely conceptual, but practical, actionable, and directly applicable to interdisciplinary teamwork.

Third, our approach shows both alignment with and represents a novel extension of the strategy for integrating disciplinary contributions. Bergmann et al. (2012) suggest that developing a CF is best achieved through a systematic approach where disciplinary-specific concepts are decontextualized and then re-contextualized within an integrated framework. The TREBRIDGE project similarly decontextualized key SES concepts from individual disciplines and refined them through collaborative feedback sessions. Moreover, the TREBRIDGE team went further by conducting comparative analyses with specific WPs, a step typically not included in the existing literature. It represents a divergence from standard practice by explicitly verifying and testing the framework in multiple contexts, thereby strengthening its robustness as a boundary object.

Finally, our experience highlights a common challenge in interdisciplinary CFs: finding the right balance between integrative breadth and disciplinary depth. As discussed by Bergmann et al. (2012) and Frodeman et al. (2017), there is an inherent tension between covering a broad range of disciplinary perspectives and maintaining detailed disciplinary rigor. In the development of the CF, the TREBRIDGE project faced similar challenges, as some team members felt that the CF leaned too heavily toward social science, while natural science aspects such as watershed dynamics should have been elaborated in more detail. Thus, integrating broad perspectives may risk oversimplifying or marginalizing discipline-specific details. The TREBRIDGE CF addresses this by incorporating direct feedback from natural and social scientists to refine discipline-specific representations. However, the adaptable CF ultimately emphasizes integrative breadth over disciplinary depth, suggesting that interdisciplinary CFs may inherently prioritize coherence over exhaustive detail. This finding aligns with Pohl et al. (2021), who noted that interdisciplinary projects often require pragmatic trade-offs between achieving comprehensive depth and creating a usable boundary object. Recognizing this trade-off is important for teams undertaking similar CF developments, so they can deliberately decide where on the spectrum of breadth vs. depth their project’s needs lie.

### Broader values and limitations

The structured approach for developing a CF that was elaborated using TREBRIDGE as a case study offers a replicable process for interdisciplinary teams, particularly those tackling complex SES problems. By establishing a shared language, incorporating continuous feedback, and viewing frameworks as dynamic practical tools, the TREBRIDGE approach promotes communication, collaboration, and integration in interdisciplinary research. This flexible process helps teams respond to evolving project needs, thereby enabling more robust, integrated research outcomes that are crucial for sustainable management and resilience planning in environmental projects. The application of this approach in TREBRIDGE highlights the importance of CFs, which serve as adaptable yet cohesive practical tools for knowledge integration.

While the collaborative and iterative CF development process offers many benefits, it also has limitations, particularly in terms of resource requirements and its focus on interdisciplinary (but not transdisciplinary) integration. First, developing an adaptable CF through a collaborative and iterative process can be time consuming and resource intensive. Projects with tight deadlines or limited resources may find it challenging to fully engage in it, thus potentially compromising the depth and/or breath and inclusivity of the CF. Rapidly evolving projects or those with urgent outcomes may require a more streamlined approach that sacrifices some of the adaptiveness of our structured approach. Second, the approach was developed and tested within a single case study, the TREBRIDGE project. While it demonstrates potential, its applicability to other contexts or disciplines remains unproven. Diverse projects with different geographic, thematic, or stakeholder dynamics may encounter challenges that this approach does not address, thus limiting its broader applicability. However, the approach is designed to be flexible—meaning that research teams are not expected to follow all ten steps or three phases exactly as outlined but should instead adapt the process to their specific context, resources, and needs. Finally, as stakeholders from policy and practice were not involved in the iterative development of the CF, the approach has so far only been tested for fostering interdisciplinary integration. This limitation may hinder its utility in projects that require significant contributions from a variety of stakeholders.

### Recommendations for developing future conceptual frameworks

Here, drawing on insights from the survey conducted in step 6, we discuss key challenges encountered during the CF development process, reflect on these experiences, and propose practical recommendations for future CF development. In particular, we recommend the following:

- Determining the right timing for initiating the process of CF development, i.e., finding the balance between starting too early and risking broadness or starting too late and risking misalignment with individual research directions: We recommend establishing a timeline aligned with project milestones and individual research schedules, including checkpoints for evaluating insights and adjusting the pace at which the CF develops.
- Selecting integrative terms and concepts as building blocks for the framework: We suggest involving a diverse team to enhance their validity and reliability, considering various perspectives from different work packages (e.g., WP3’s focus on “biophysical pressure” and WP2’s emphasis on “actors” and “resources”).
- Integrating the perspectives into a cohesive framework: We recommend regular interdisciplinary team meetings and data triangulation through group interviews, workshops, and surveys to facilitate this process and adapt it, if necessary.
- Managing time: We suggest allocating sufficient time for each step, including reflection and revision in step 7 and 10, to ensure a thorough development and high-quality interdisciplinary collaboration and integration.
- Communicating added value: We recommend explicitly and repeatedly emphasizing the added value of each step and the CF itself to research colleagues to prevent them from failing to see *“how this [CF] can guide us in the research”* as one TREBRIDGE researcher stated.

## Conclusion

The primary contribution of this article is the structured approach and the iterative and collaborative process used to develop a CF as a boundary object. This process facilitates the integration of knowledge from various disciplines, enabling the construction of a flexible framework that can accommodate the dynamics of different SES. It offers a starting point for other interdisciplinary projects, providing a replicable process for developing and using conceptual frameworks in diverse SES contexts and beyond. Rather than offering a conclusive solution to or replacement for existing frameworks, the process is intended to inspire adaptation and refinement in interdisciplinary research, highlighting the necessity of flexibility when addressing complex socio-ecological challenges. Importantly, our proposed approach emphasizes iterative engagement with the boundary object, ensuring that the CF evolves over time and remains responsive to empirical insights as well as adaptable to changes in the research project itself. The CF developed by the TREBRIDGE team exemplifies the power of this approach, offering a practical, responsive framework for SES research in complex Alpine ecosystems. Thus, the novelty of this paper lies not in introducing new methods and procedures, but in providing a structured, replicable approach (three phases with ten steps) that is described in detail, thus filling a gap in practical guidance for interdisciplinary teams.

In addition to the findings and contributions discussed above, several aspects merit further exploration and elaboration in future research. Future application of this approach in diverse SES contexts could extend its relevance, inspiring further adaptations and driving the examination of challenges and opportunities across interdisciplinary research landscapes and beyond. Including practitioners and other stakeholders could strengthen the CF by incorporating the transdisciplinary aspect into the process of CF development. The collaborative and iterative nature of the process (see Fig. 1) could therefore be expanded to include an additional level, i.e., the stakeholders. Additionally, further research could examine how such CFs can influence activities outside the research project itself, such as informing teaching activities and making it more holistic and inclusive. It is crucial to acknowledge that the application of our CF approach to other projects is likely to raise new questions and concerns due to different perspectives. Thus, additional steps may be required to reconcile differences, such as facilitating discussions to identify commonalities or refining the framework to accommodate diverse perspectives. After all, we do not claim to have found a holistic solution that is applicable across the board, but we rather wish to provide an additional puzzle piece with our structured and flexible approach that can help to better understand interdisciplinary connections and thus support an increase in the resilience of SES through dedicated research. Lastly, our work intends to inspire and facilitate the development and use of CFs in diverse research contexts, thereby fostering critical reflection and continuous adaptation of our structured approach for inter- and transdisciplinary research. Ultimately, we are convinced that this approach and its multi-step process have the potential to generate new knowledge that can effectively address the complex socio-ecological challenges we face by supporting interdisciplinary communication, collaboration, and integration.

## Supplementary Information

Below is the link to the electronic supplementary material.

Supplementary file1 (PDF 983 KB)

## References

[CR1] Angelstam, P., K. Andersson, M. Annerstedt, R. Axelsson, M. Elbakidze, P. Garrido, P. Grahn, K. I. Jönsson, et al. 2013. Solving problems in social-ecological systems: Definition, practice and barriers of transdisciplinary research. *Ambio* 42: 254–265. 10.1007/s13280-012-0372-4. 23475660 10.1007/s13280-012-0372-4 PMC3593036

[CR2] Barnes, B. 2013. *Scientific knowledge and sociological theory*. Routledge. 10.4324/9780203706541.

[CR3] Beisner, B. E., D. T. Haydon, and K. Cuddington. 2003. Alternative stable states in ecology. *Frontiers in Ecology and the Environment* 1: 376–382. 10.1890/1540-9295(2003)001[0376:ASSIE]2.0.CO;2.

[CR4] Bergmann, M., T. Jahn, T. Knobloch, W. Krohn, and C. Pohl. 2012. *Methods for transdisciplinary research: A primer for practice*. Campus Verlag.

[CR5] Berkes, F., J. Colding, and C. Folke. 2008. *Navigating social-ecological systems: Building resilience for complexity and change*. Cambridge University Press.

[CR6] Bogen, J. 2009. Theory and observation in science. In *Stanford Encyclopedia of Philosophy* (Vol. 33, pp. 1–23).

[CR7] Castellví, M. T. C. 2003. Theories of terminology: Their description, prescription and explanation. *Terminology. International Journal of Theoretical and Applied Issues in Specialized Communication* 9: 163–199. 10.1075/term.9.2.03cab.

[CR8] Clark, W. C., T. P. Tomich, M. van Noordwijk, D. Guston, D. Catacutan, N. M. Dickson, and E. McNie. 2016. Boundary work for sustainable development: Natural resource management at the consultative group on international agricultural research (CGIAR). *Proceedings of the National Academy of Sciences of the United States of America* 113: 4615–4622. 10.1073/pnas.0900231108. 10.1073/pnas.0900231108 PMC4855572 21844351

[CR9] Costanza, R., and S. E. Jorgensen. 2002. *Understanding and solving environmental problems in the 21st century: Toward a new, integrated hard problem science*. Elsevier. 10.1016/B978-0-08-044111-5.X5000-8.

[CR10] Dick, M., A. M. Rous, V. M. Nguyen, and S. J. Cooke. 2016. Necessary but challenging: Multiple disciplinary approaches to solving conservation problems. *Facets* 1: 67–82. 10.1139/facets-2016-0003.

[CR11] Dölker, J. 2022. *Literature review: Assessing impacts of changes in the trophic state and cyanobacteria blooms on a local economy: Western lake Erie as a case study*. University of Geneva.

[CR12] Folke, C., R. Biggs, A. V. Norström, B. Reyers, and J. Rockström. 2016. Social-ecological resilience and biosphere-based sustainability science. *Ecology and Society* 21: 16. 10.5751/ES-08748-210341.

[CR13] Freeth, R., and G. Caniglia. 2020. Learning to collaborate while collaborating: Advancing interdisciplinary sustainability research. *Sustainability Science* 15: 247–261. 10.1007/s11625-019-00701-z.

[CR14] Frodeman, R., J. T. Klein, and R. C. D. S. Pacheco. 2017. *The Oxford handbook of interdisciplinarity*. Oxford University Press. 10.1093/oxfordhb/9780198733522.001.0001.

[CR15] Graff, H. J. 2016. The “problem” of interdisciplinarity in theory, practice, and history. *Social Science History* 40: 775–803. 10.1017/ssh.2016.31.

[CR16] Hadorn, G. H., H. Hoffmann-Riem, S. Biber-Klemm, W. Grossenbacher-Mansuy, D. Joye, C. Pohl, U. Wiesmann, and E. Zemp. 2008. *Handbook of transdisciplinary research*, vol. 10. Springer. 10.1007/978-1-4020-6699-3.

[CR17] Hertz, T., and M. Schlüter. 2015. The SES-Framework as boundary object to address theory orientation in social–ecological system research: The SES-TheOr approach. *Ecological Economics* 116: 12–24. 10.1016/j.ecolecon.2015.03.022.

[CR18] Hicks, C. C., C. Fitzsimmons, and N. V. Polunin. 2010. Interdisciplinarity in the environmental sciences: Barriers and frontiers. *Environmental Conservation* 37: 464–477. 10.1007/978-1-4020-6699-3.

[CR19] Hoffmann, S. 2024. Synthesizing. In *Elgar encyclopedia of interdisciplinarity and transdisciplinarity*, ed. F. Darbellay, 505–508. Edward Elgar Publishing. 10.4337/9781035317967.ch111.

[CR20] Hoffmann, S., C. Pohl, and J.G. Hering. 2017. Methods and procedures of transdisciplinary knowledge integration: Empirical insights from four thematic synthesis processes. *Ecology and Society* 22: 27. 10.5751/es-08955-220127.

[CR21] Hoffmann, S., J. Thompson Klein, and C. Pohl. 2019. Linking transdisciplinary research projects with science and practice at large: Introducing insights from knowledge utilization. *Environmental Science & Policy* 102: 36–42. 10.1016/j.envsci.2019.08.011.

[CR22] Hölscher, K., J. M. Wittmayer, and D. Loorbach. 2018. Transition versus transformation: What’s the difference? *Environmental Innovation and Societal Transitions* 27: 1–3. 10.1016/j.eist.2017.10.007.

[CR23] Jessop, B., and N.-L. Sum. 2001. Pre-disciplinary and post-disciplinary perspectives. *New Political Economy* 6: 89–101. 10.1080/13563460020027777.

[CR24] Krütli, P., M. Stauffacher, T. Flüeler, and R. W. Scholz. 2010. Functional-dynamic public participation in technological decision-making: Site selection processes of nuclear waste repositories. *Journal of Risk Research* 13: 861–875. 10.1080/13669871003703252.

[CR25] Leavy, P. 2016. *Essentials of transdisciplinary research: Using problem-centered methodologies*. Routledge. 10.4324/9781315429137.

[CR26] Ledesma, J. 2014. Conceptual frameworks and research models on resilience in leadership. *SAGE Open* 4: 215824401454546. 10.1177/2158244014545464.

[CR27] Lieberherr, E., J. Dölker, H. Salomon, V. Schick, I. Logar, H. Bugmann, F. Schlunegger, L. König, et al. 2025. Science integration and a participatory scenario process. An inter- and transdisciplinary study from the Alps. *GAIA Ecological Perspectives for Science and Society* 34: 35–41. 10.14512/gaia.34.1.4

[CR28] Lieberherr, E., H. Bugmann, S. Hoffmann, and F. Schlunegger. 2021. *Transformation toward resilient ecosystems: bridging natural and social sciences (TREBRIDGE)* [Proposal for SNSF Sinergia Project].

[CR29] Liu, J., T. Dietz, S. R. Carpenter, M. Alberti, C. Folke, E. Moran, A. N. Pell, P. Deadman, et al. 2007. Complexity of coupled human and natural systems. *Science* 317: 1513–1516. 10.1126/science.1144004. 17872436 10.1126/science.1144004

[CR30] Menken, S. and M. Keestra. 2016. An introduction to interdisciplinary research: Theory and practice. *An Introduction to Interdisciplinary Research*, pp. 1–128. 10.1515/9789048531615.

[CR31] Mollinga, P. P. 2010. Boundary work and the complexity of natural resources management. *Crop Science* 50: S-1–S-9. 10.2135/cropsci2009.10.0570.

[CR32] Nurius, P. S. and S. P. Kemp. 2019. Individual-level competencies for team collaboration with cross-disciplinary researchers and stakeholders. *Strategies for team science success: Handbook of evidence-based principles for cross-disciplinary science and practical lessons learned from health researchers*, pp. 171–187. 10.1007/978-3-030-20992-6_13.

[CR33] O’Rourke, M. 2017. Comparing methods for cross-disciplinary research. In *The Oxford handbook of interdisciplinarity*, 2nd ed., ed. R. Frodeman, 276–290. Oxford University Press. 10.1093/oxfordhb/9780198733522.013.23.

[CR34] O’Rourke, M., S. Crowley, and C. Gonnerman. 2016. On the nature of cross-disciplinary integration: A philosophical framework. *Studies in History and Philosophy of Science Part C Studies in History and Philosophy of Biological and Biomedical Sciences* 56: 62–70. 10.1016/j.shpsc.2015.10.003. 10.1016/j.shpsc.2015.10.003 26601600

[CR35] Ostrom, E. 2007. A diagnostic approach for going beyond panaceas. *Proceedings of the National Academy of Sciences* 104: 15181–15187. 10.1073/pnas.0702288104. 10.1073/pnas.0702288104 PMC2000497 17881578

[CR36] Ostrom, E. 2009. A general framework for analyzing sustainability of social-ecological systems. *Science* 325: 419–422. 10.1126/science.1172133. 19628857 10.1126/science.1172133

[CR37] Pennington, D., S. Vincent, D. Gosselin, and K. Thompson. 2021. Learning across disciplines in socio-environmental problem framing. *Socio-Environmental Systems Modelling* 3: 17895. 10.18174/sesmo.2021a17895.

[CR38] Plavén-Sigray, P., G. J. Matheson, B. C. Schiffler, and W. H. Thompson. 2017. The readability of scientific texts is decreasing over time. *eLife* 6: e27725. 10.7554/eLife.27725. 28873054 10.7554/eLife.27725 PMC5584989

[CR39] Pohl, C., and G. H. Hadorn. 2008. Methodological challenges of transdisciplinary research. *Natures Sciences Sociétés* 16: 111–121. 10.1051/nss:2008035.

[CR40] Pohl, C., and G. H. Hadorn. 2017. Frameworks for transdisciplinary research: Framework #1. *GAIA Ecological Perspectives for Science and Society* 26: 232–232. 10.14512/gaia.26.3.3.

[CR41] Pohl, C., J. T. Klein, S. Hoffmann, C. Mitchell, and D. Fam. 2021. Conceptualising transdisciplinary integration as a multidimensional interactive process. *Environmental Science and Policy* 118: 18–26. 10.1016/j.envsci.2020.12.005.

[CR42] Pohl, C. 2022. *Three types of knowledge tool*.

[CR43] ProClim, C. 1997. Research on sustainability and global change—visions in science policy by swiss researchers. ProClim—Forum for Climate and Global Change and Swiss Academy of Sciences, Bern.

[CR44] Raymond, C. M., I. Fazey, M. S. Reed, L. C. Stringer, G. M. Robinson, and A. C. Evely. 2010. Integrating local and scientific knowledge for environmental management. *Journal of Environmental Management* 91: 1766–1777. 10.1016/j.jenvman.2010.03.023. 20413210 10.1016/j.jenvman.2010.03.023

[CR45] Rossini, F. A., and A. L. Porter. 1979. Frameworks for integrating interdisciplinary research. *Research Policy* 8: 70–79. 10.1016/0048-7333(79)90030-1.

[CR46] Salomon, H. 2023. *Toward resilient ecosystems: An analysis of the opportunities and challenges of science integration and researcher’s roles.* Master’s Thesis, ETH Zürich.

[CR47] Schäpke, N., F. Stelzer, G. Caniglia, M. Bergmann, M. Wanner, M. Singer-Brodowski, D. Loorbach, P. Olsson, et al. 2018. Jointly experimenting for transformation? Shaping real-world laboratories by comparing them. *GAIA-Ecological Perspectives for Science and Society* 27: 85–96. 10.14512/gaia.27.S1.16.

[CR48] Scheffer, M., S. Carpenter, J. A. Foley, C. Folke, and B. Walker. 2001. Catastrophic shifts in ecosystems. *Nature* 413: 591–596. 10.1038/35098000. 11595939 10.1038/35098000

[CR49] Star, S. L., and J. R. Griesemer. 1989. Institutional ecology, ‘translations’ and boundary objects: Amateurs and professionals in Berkeley’s museum of vertebrate zoology, 1907–39. *Social Studies of Science* 19: 387–420. 10.1177/030631289019003001.

[CR50] Tengo, M., E. S. Brondizio, T. Elmqvist, P. Malmer, and M. Spierenburg. 2014. Connecting diverse knowledge systems for enhanced ecosystem governance: The multiple evidence base approach. *Ambio* 43: 579–591. 10.1007/s13280-014-0501-3. 24659474 10.1007/s13280-014-0501-3 PMC4132468

[CR51] Tobi, H., and J. K. Kampen. 2018. Research design: The methodology for interdisciplinary research framework. *Quality and Quantity* 52: 1209–1225. 10.1007/s11135-017-0513-8. 29674791 10.1007/s11135-017-0513-8 PMC5897493

[CR52] Turnhout, E. 2019. Interdisciplinarity and the challenge of knowledge integration. In *Environmental expertise: Connecting science, policy and society*, ed. E. Turnhout, W. Halffman, and W. Tuinstra, 152–164. Cambridge University Press. 10.1017/9781316162514.013.

[CR53] Van der Steen, M., and M. Van Twist. 2013. Foresight and long-term policy-making: An analysis of anticipatory boundary work in policy organizations in The Netherlands. *Futures* 54: 34. 10.1016/j.futures.2013.09.009.

[CR54] Virapongse, A., S. Brooks, E. C. Metcalf, M. Zedalis, J. Gosz, A. Kliskey, and L. Alessa. 2016. A social-ecological systems approach for environmental management. *Journal of Environmental Management* 178: 83–91. 10.1016/j.jenvman.2016.02.028. 27131638 10.1016/j.jenvman.2016.02.028

