# Supplementary material for: Developing a conceptual framework for interdisciplinary communication, collaboration, and integration: A structured approach
Source: Ambio. 2025 Aug 20;54(12):2118–34. doi: 10.1007/s13280-025-02210-z (PMC12569237; doi:10.1007/s13280-025-02210-z)
Supplement: Supplementary file 1 — Supplementary file1 (PDF 983 KB) [file 13280_2025_2210_MOESM1_ESM.pdf]

Ambio

Supplementary Information

This supplementary information has not been peer reviewed.

Title: Developing a conceptual framework for interdisciplinary communication, collaboration, and integration: a structured approach

Appendix A. Example of definitions for the term ‘transformation’ (step 2)

Transformation	The change of a system from one state (where we are) to another state that is more desirable (where we want to be).	Pohl, C., 2022. Three types of knowledge tool. td-net toolbox profile (19).Swiss Academies of Arts and Sciences: td-net toolbox for co-producing knowledge. transdisciplinarity.ch/toolbox. DOI 10.5281/zenodo.7015070
Transformation	Large-scale societal change, often involving a fundamental shift in the interaction between humans and their environment.	Hölscher K, Wittmayer JM, Loorbach D (2018) Transition versus transformation: What’s the difference? Environmental Innovation and Societal Transitions 27:1-3. doi:10.1016/j.eist.2017.10.007
Transformation/ Transition	Research approaches concerned with global environmental change, such as resilience (Olsson et al., 2014) and transformative adaptation (O’Brien, 2012), adopted ‘transformation’ to refer to fundamental shifts in human and environmental interactions and feedbacks. (Hölscher et al. 2018)<br>Both concepts are often associated with normative notions to describe the desirability of transition and transformation. The unsustainability of current societal systems is contrasted with a collectively defined sustainability orientation for desirable transitions and transformations (Loorbach et al., 2017; Olsson et al., 2014). Transformation is additionally applied in relation to concepts such as resilience and planetary boundaries, which support an assessment of potentially detrimental implications of undesirable transformations and orient desirable transformations towards ‘safe and just operating spaces’ (Folke et al., 2010; Raworth, 2012). (Hölscher et al. 2018)<br>Besides these normative notions, transformation is also used as analytical concept or for strategic purposes. Concepts that use transformation in a more strategic way provide ways of dealing with problems and crises that are assumed to be effective and socially desirable. An analytical concept of transformation focuses on understanding the different transformative dynamics and to create insights into the obstacles facing certain strategic ways of dealing with problems and crises. Furthermore, it involves analysing the contexts and barriers in addition to real and potential systemic changes (Brand 2016)	Hölscher K, Wittmayer JM, Loorbach D (2018) Transition versus transformation: What’s the difference? Environmental Innovation and Societal Transitions 27:1-3. doi:10.1016/j.eist.2017.10.007<br><br>Brand, U. 2016. “Transformation” as a new critical orthodoxy. The strategic use of the term “transformation” does not prevent multiple crises. GAIA 25/1: 23 – 27.
Transformation	Transformation of a system refers to a process altering fundamental aspects of a system, such as its dynamics, function, etc., which can occur both because of endo- and exogenous changes, i.e. a change of its dynamics and independent variables change.	Beisner, B. E. (2012) ‘Alternative stable states’, Nature Education Knowledge 3(10):33<br>Available at:<br>https://www.nature.com/scitable/knowledge/library/alternative-stable-states-

	Scenario planning is the process of building a framework in order to envision plausible transformations and help social decision processes (Peterson et al. 2003), therefore, depending on the chosen context factors, also influencing the transformability of the system.<br>Transformation occurs in systems when the existing system (structure, function, etc.) is untenable, due to changes in exogenous, endogenous variables or both. It can be triggered e.g. by a shift of the system's state.<br>The state of an ecosystem or a community is determined by the interaction between biotic and abiotic elements, which are interdependent at different spatial and temporal scales (Beisner, 2012; Beisner et al, 2003). A state can be called stable, when processes and structures of a system are mutually reinforcing and the considered system tends to come back to a stable equilibrium point (or stable limit cycle). A state is party defined by the shape of its basin of attraction (cf Resilience).<br>Some systems are known to exhibit several possible stable states in which they can be found and shifts between states can be observed. According to Scheffer and Carpenter (2003), ecosystems respond to changes in parameters, either rather linearly, profoundly when a critical level of change is reached or abruptly showing hysteresis, when passing a tipping point. After a threshold (or tipping point) is passed, the dynamics of the system accelerate dramatically and a fundamental change in the state of the system occurs. If the new state is untenable, system dynamics can lead to processes of adaptation and/or transformation.	78274277/<br><br>Beisner, B. E., et al. (2003). "Alternative stable states in ecology." Frontiers in Ecology and the Environment **1**(7): 376-382.<br><br>Elmqvist, T., Andersson, E. Frantzeskaki, N., McPhearson, T., Olsson, P., Gaffney, O., Takeuchi, K., Folke, C. (2019). Sustainability and resilience for transformation in the urban century. *Nature Sustainability* 2: 267–273.<br><br>Holling, C. S. (1973). "Resilience and stability of ecological systems." Annu. Rev. Ecol. Syst. **4**: 1-23.<br><br>Scheffer, M. and S. R. Carpenter (2003). "Catastrophic regime shifts in ecosystems: linking theory to observation." Trends in Ecology & Evolution **18**(12): 648-656.<br><br>Peterson, G. D., et al. (2003). "Scenario Planning: a Tool for Conservation in an Uncertain World." Conservation Biology **17**(2): 358-366.<br><br>van Nes, E. H., et al. (2016). "What Do You Mean, 'Tipping Point'?" Trends Ecol Evol **31**(12): 902-904.<br><br>Walker, B., et al. (2004). "Resilience, Adaptability and Transformability in Social-ecological Systems." Ecology and Society **9**(2).

Compilation of the definitions for the interdisciplinary term ‘transformation’ from integration leaders (step 2) that was used for discussion among TREBRIDEGE researchers in step 3.

a) **Transformation** refers to a **large-scale radical, non-linear and structural change** in **complex adaptive systems** such as **society**. It can occur both because of **endo- and exogenous changes**. It often involves a **fundamental shift in the interaction** between humans and their environment. Transformation occurs in systems when the existing system (structure, function, etc.) is untenable, due to changes in exogenous, endogenous variables or both. It can be triggered e.g. by a shift of the system’s state.	Does everybody agree?<br>Does anyone want to add anything/change the definition?<br><br>Do you agree with the three dimensions (normative, analytical, strategic) of the transformation concept?<br><br>What do these three dimensions mean for TREBRIDGE? Which dimensions do we consider?
b) **Transformation** is applied in **relation to concepts** such as **resilience and planetary boundaries**, which support an assessment of potentially detrimental implications of **undesirable transformations** and orient **desirable transformations** towards ‘safe and just operating spaces’	
c) Transformation is often associated with **normative notions** to describe the desirable transformation of a system from **one state** (where we are) **to another state that is more desirable** (where we want to be). Besides this normative notion, transformation is also used as an analytical concept or for strategic purposes (Brand 2016). An **analytical concept** of transformation focuses on understanding the different transformative dynamics and to create insights into the obstacles facing certain strategic ways of dealing with problems and crises. Furthermore, it involves analyzing the contexts and barriers in addition to real and potential systemic changes. A **strategic concept** of transformation provides ways of dealing with problems and crises that are assumed to be effective and socially desirable	

Output of discussion about the definition of the term ‘transformation’ among TREBRIDGE researchers (step 3).

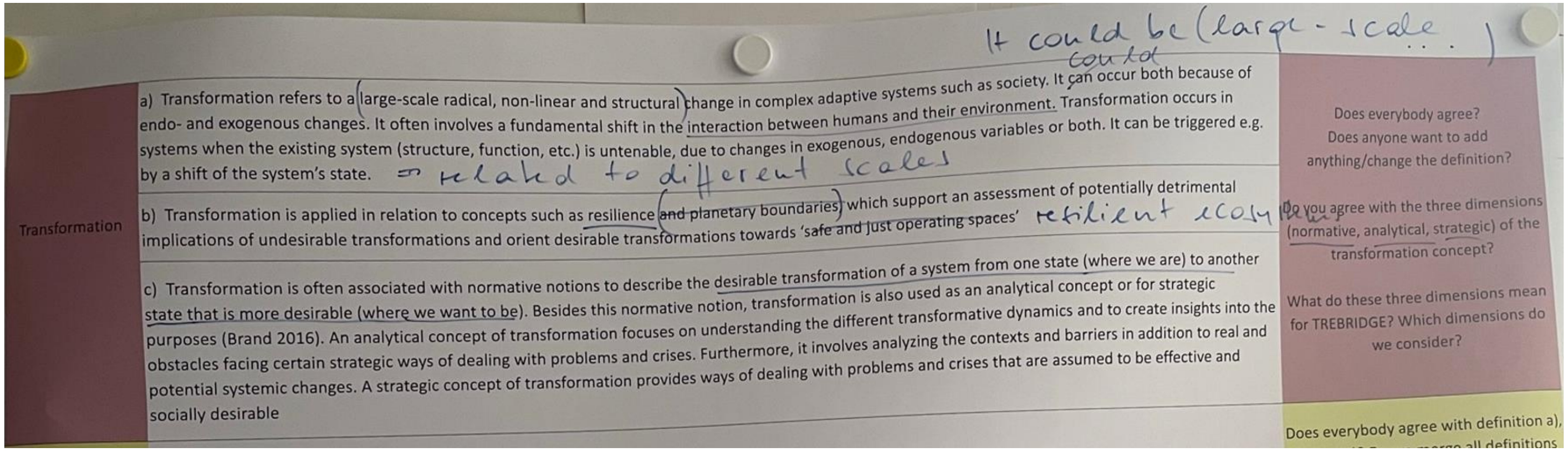

Appendix B. Detailed definition and description of clustered boundary concepts (step 4)

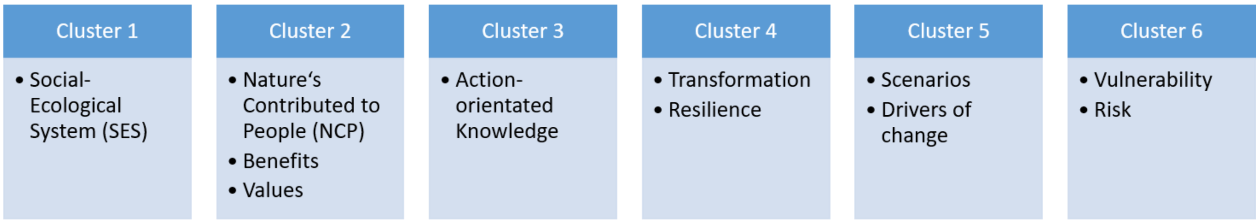

Figure 1a: Overview of the six clusters grouping the key boundary concepts for the CSF. The detailed definitions and descriptions can be found in Appendix A.

Cluster 1: Socio-Ecological Systems (SES)

Cluster 1 comprises concepts and frameworks that examine the interactions between social and ecological systems. Its primary focus is on comprehending the complex interdependencies and interconnections between various systems, such as social and ecological systems, and how changes in one system can reverberate through the other. The project recognizes that alpine ecosystems are interconnected and interdependent systems that are shaped by socio-political, environmental, and economic factors. This cluster can potentially provide a foundation for understanding the intricate relationships among these factors and organizing them based on the relevant dimensions of the system.

Social-Ecological System: A SES is defined as integrated complex systems in which people interact with natural components and are an intrinsic part of nature (Liu et al. 2007; Ostrom 2007). By employing the SES approach, it becomes possible to consider social, biophysical, and linking variables, such as Nature's Contributions to People (NCPs), and integrate various methodologies from diverse fields to assess intricate interactions within the system. Within such systems, critical transition points, such as tipping points and nonlinearity, may emerge, leading to shifts from one state to another over time or space.

Cluster 2: Nature's Contributions to People (NCP)

The project acknowledges the vital role of alpine ecosystems in providing a diverse range of Nature's Contributions to People (NCPs) that are essential for human well-being. Cluster 2 focuses on understanding the origins of NCPs, highlighting the significance of comprehending the trade-offs and synergies among different NCPs, and integrating the NCP concept into decision-making processes.

Nature's Contributions to People (NCP): The term NCP is defined as all the contributions, both positive and negative, biosphere and non-biosphere processes (i.e., biodiversity and erosion) to people's quality of life. NCPs can have beneficial effects, such as food provision, water purification, flood control, and artistic inspiration. However, they can also have detrimental impacts, such as

disease transmission or predation, that harms individuals or their assets. The perception, cultural and socioeconomic context, spatial and temporal considerations, as well as the specific aims of a study, influence whether an NCP is perceived as beneficial or detrimental (Díaz et al. 2018). Compared with the concept of Ecosystem Service, the NCP concept gives a more holistic assessment of values of nature. While Ecosystem Services are a critical part of NCP, it encompasses a broader perspective that recognizes additional ways in which nature contributes to human well-being, including cultural values and social relations (Ellis, Pascual, and Mertz 2019).

Diaz et al (2018) identified 18 different reporting categories of NCPs, which can be classified into three groups:

- Regulating (e.g., regulation of air quality, regulation of climate, regulation of ocean acidification, etc.)
- Non-material (e.g., learning and inspiration, physical and psychological experiences, etc.)
- Material (e.g., energy, food, and feed, etc.)

Cluster 3: Action-Oriented Knowledge

Cluster 3 encompasses a set of concepts and practices that aim to address environmental, social, and economic knowledge of natural systems and processes that can be used to facilitate sustainable solutions and system transformation.

Action-Oriented Knowledge: The term action-oriented knowledge refers to knowledge that provides guidance on taking effective action to address challenges, solve problems, or achieve certain goals (Caniglia et al. 2020). Specifically, within the context of sustainability and resilience of Social-Ecological Systems (SESs), action-oriented knowledge encompasses insights on how to foster transformative change towards sustainability by integrating diverse forms of knowledge. It involves actively working with multiple knowledge types to develop integrated solutions, which encompass not only scientific knowledge but also empirical knowledge from stakeholders. Moreover, action-oriented knowledge also shapes the identification and prioritization of suitable solution options, considering the complex system characteristics inherent in SESs.

Cluster 4: Transformation and Resilience

Cluster 4 provides insights into how to promote transformative change towards more sustainable and resilient systems. It emphasizes the importance of understanding the potential for transformation, including both desirable and undesirable outcomes. The framework also emphasizes the importance of understanding the factors that influence the resilience of a system, including natural hazards, land use practices, and governance arrangements.

Transformation: Transformation refers to the fundamental changes that systems undergo in response to external pressures or internal dynamics. Such change could be large-scale, radical, non-linear and/or structural. Transformations can be triggered by both endogenous (internal) and exogenous (external) factors, and often involve a fundamental shift in the relationship between humans and their environment. In the specific context of alpine ecosystems, various factors contribute to their

transformation. Endogenous factors encompass geological processes like erosion and tectonic activity, as well as ecological changes such as shifts in species composition and vegetation dynamics. Exogenous factors, including climate change, land use changes, and human activities like tourism and resource extraction, exert external pressures that further drive the transformation of alpine ecosystems.

Resilience: Resilience is the capacity of a system, community, or society to absorb, adapt to, recover from, or accommodate the effects of disturbances or slow onset events (such as climate change or droughts) in a timely, efficient, and ecological manner. It encompasses not only the ability to endure and bounce back from disruptions but also the preservation and restoration of ecological quality, including maintaining biodiversity, ecosystem function, and ecological integrity. Resilience extends to multiple dimensions, encompassing the degree, manner, and pace of recovery. It necessitates proactive risk management to safeguard essential structures and functions. In the context of TREBRIDGE, we define resilient ecosystems as natural systems, such as watersheds, that possess the ability to absorb changes or recover quickly from stressed conditions, including sudden events like landslides and floods, as well as slow onset events such as climate change and droughts.

Cluster 5: Scenarios and Drivers of Change

Cluster 5 refers to a group of interconnected concepts that are commonly used to analyze and prepare for future situations but also for risk management and policy development. This project recognizes that alpine ecosystems are undergoing rapid and complex changes due to a range of environmental, social, economic, and political factors. The project also recognizes the importance of developing scenarios to explore different futures for alpine ecosystems. By considering multiple scenarios and drivers of change, we can be better prepared to navigate uncertainty and make decisions that are sustainable, equitable, and aligned with our goals and values.

Scenarios: Scenarios refer to potential future pathways that are shaped by drivers of change, which can be either internal or external forces, such as climate change. These drivers of change influence various context factors, which are the characteristics of a specific place that can impact the system's ability to undergo transformation. Scenarios are conceptualized as plausible and possible futures, and they can be described through narratives or numerical representations. They provide meaningful and internally coherent alternatives to each other, enabling a comprehensive exploration of future possibilities.

Drivers of change: Drivers of change encompass both endogenous and exogenous forces that exert pressure on a study region, leading to changes within complex adaptive systems. These drivers are thus closely associated with the concept of transformations. Examples of drivers of change include technological advancements, the right to protection, droughts, flooding, and other factors that influence the system dynamics and its capacity to adapt and transform over time.

Cluster 6: Vulnerability, Risk, Benefits, and Values

Cluster 6 refers to a group of interconnected concepts that are commonly considered when making decisions and describing a system in a particular action or situation. While vulnerability, risk, benefits, and values can be applied to any human or non-human system, they primarily adopt a human-centric perspective to assess and evaluate systems. This cluster holds relevance for governance decisions and the generation of action-oriented knowledge.

Risk: Risk refers to the probability of an outcome having a negative effect on a social-ecological system. It is typically determined by considering the combined effects of hazards, the exposure of assets or individuals to those hazards, and the vulnerability of the exposed elements. Include natural hazards.

Vulnerability: Vulnerability relates to the characteristics and circumstances of a social-ecological system, that make it susceptible to the damaging effects of a hazard. Vulnerability stems from a range of physical, social, economic, and environmental factors. It varies within a social-ecological system, and evolves over time, independent of its exposure to hazards. A system's vulnerability can influence its resilience, and vice versa. High vulnerability can undermine resilience, making it more challenging for a system to withstand and recover from hazards. Conversely, resilience-building efforts can help reduce vulnerability by enhancing adaptive capacity and addressing underlying drivers of vulnerability.

Value: Value encompasses multiple meanings, including principles associated with a given worldview or cultural context, individual preferences for specific states of the world, the importance of something for itself or others, or simply a measure. These different meanings of 'value' can intersect. For instance, ethical principles may guide the importance assigned to various aspects of nature's contributions to people (NCPs), leading to preferences for specific courses of action. Such preferences can be measured using appropriate valuation tools. It is crucial to avoid conflating these diverse meanings of value.

Benefits: Benefits represent positive changes in well-being resulting from the fulfillment of needs. The arise from both direct and indirect outputs of ecosystems, which are transformed into goods or experiences that may no longer retain functional connections to the systems from which they originated.

Appendix C. Interview guideline (step 5)

Preparation Interview

This list should help the interviewer to prepare for the Interview.

- Prepare updated Framework and definitions.
  - The updated Framework always integrates the elements discussed in the last interview.
- Material
  - White Board or Poster => one for each framework
  - Pencils, Stickers, (Magnets)
  - Recording device
  - Print out this interview guideline, the list of integrative terms and concepts, definitions (two versions), our own framework
- Send preparation to the WPs
  - Interview structure & definitions (see Appendix A for the definitions)

Interview

Following the main tasks and some mental notes to help focus on the key elements of the interview.

Introduce ourselves and explain the interview structure (5 min).

- The goal of the interviews is to develop a conceptual framework for the TREBRIDGE project that accounts for the different perspectives of the WPs.
- 3 Elements
  - Definitions
  - Developing own framework
    - Important that there is no right or wrong but exploring different perspectives.
  - Comparing and validating the framework
- Tight time schedule (90 mins in total). Therefore, we will interrupt when discussions go on for too long.
- We will record the Interview if this is all right.

Clarifying the definition (25 min)

- Describe Process: We will present you an aggregated version of some key concepts and terms that are based on the concepts/terms you discussed earlier but are simplified. The goal of this discussion is to build common understandings of each term and concepts.
  - Top 3 minutes for discussing the definition of each cluster.
- Does this definition make sense to you, or is something missing? If yes, what would you like to change and why?
  - Directly integrate it into the definition, or explain why we did not integrate it.
    - Possible explanations
      - Level of detail (is included but not explicitly mentioned)
      - Out of the scope of the research project
      - Part of another concept or term

Developing Framework (25 min)

- Explain the process: 20 minutes to develop a conceptual system framework for the TREBRIDGE project. To do so, you can use 6 clusters of terms (which can also be modified if needed, and new concepts/terms can be added) and indicated dependencies and processes linking them (developing a framework is a long-lasting iterative process. You are not expected to have a complete final product, but all the relevant terms should be included). Illustrate it all graphically using the provided material. In the end, present the framework shortly (5 minutes).
    - Clarify questions from our side if required
- Observe/Guide the process
    - Be available for questions
    - Make notes regarding similarities and differences between the frameworks
- Discussing Framework
- We present our framework (5 min)
    - While presenting, the others should, for themselves, note questions and similarities/differences.
    - Clarify questions from their side if needed.
- Now we rotate between interview team/WP and always discuss the next most important similarity/differences between the framework (starting with 1 round of similarities and afterward differences still all clarified or time is up) (20 mins)

Depending on the discussion, this structure can also be loosened.

Appendix D. Different CFs that were developed during the different steps (steps 5-10)

Step 5: CF_i

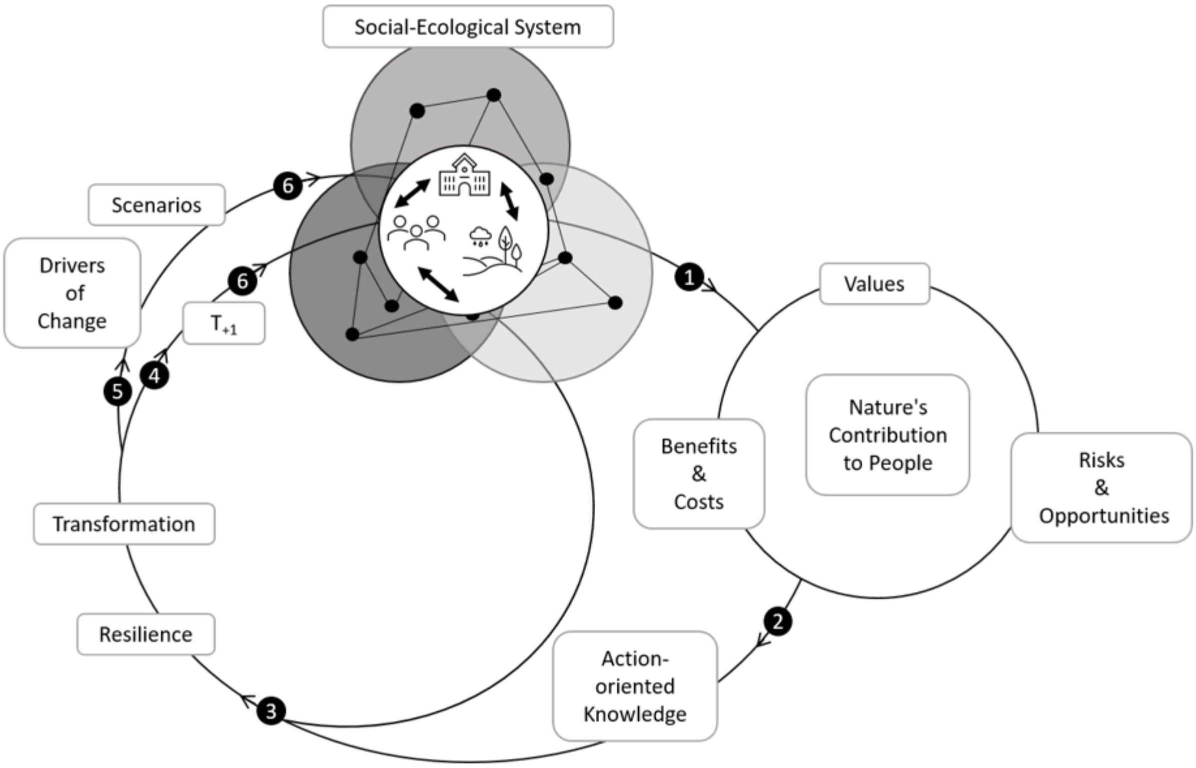

Step 6: CF_ii

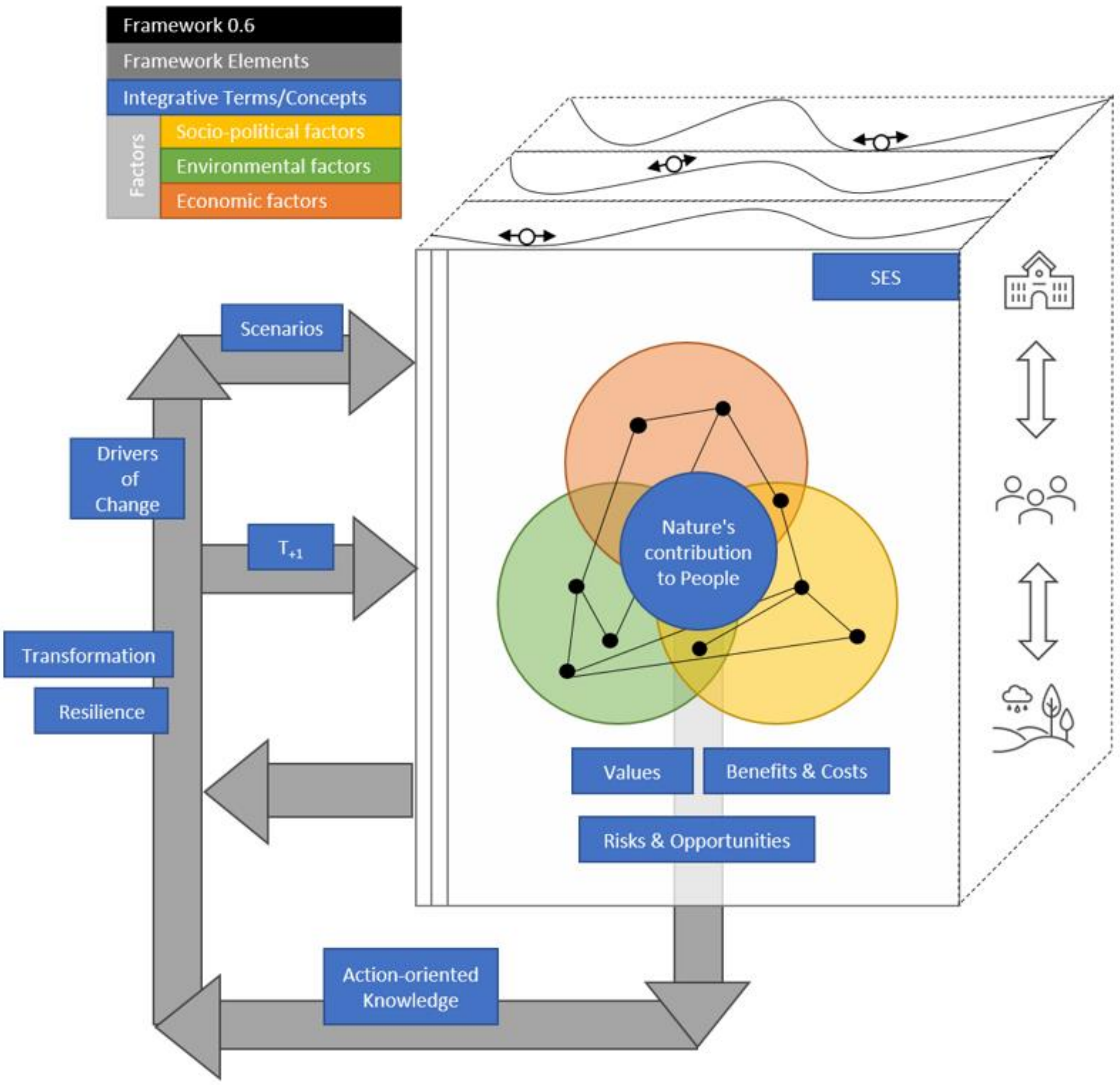
Framework 0.6
Framework Elements
Integrative Terms/Concepts
Factors
Socio-political factors
Environmental factors
Economic factors
SES
Scenarios
Drivers of Change
T+1
Transformation
Resilience
Nature's contribution to People
Values
Benefits & Costs
Risks & Opportunities
Action-oriented Knowledge

Step 7: CF_iii

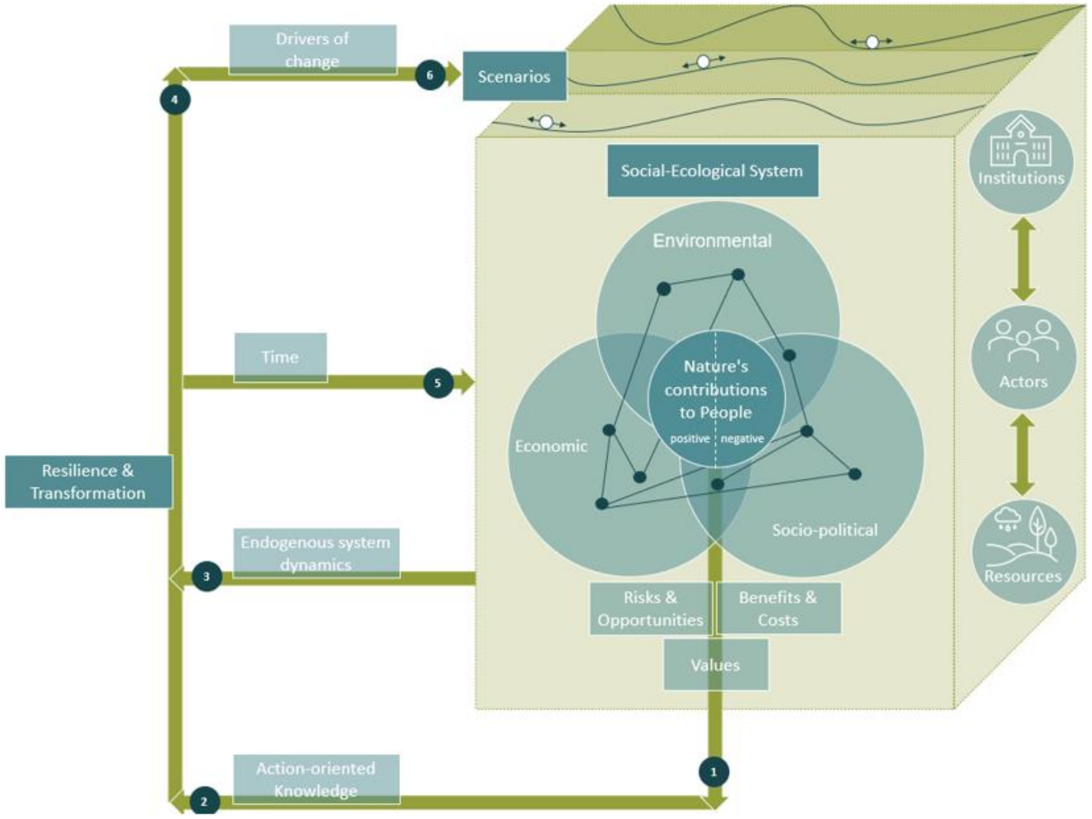

Step 8: CF_iv

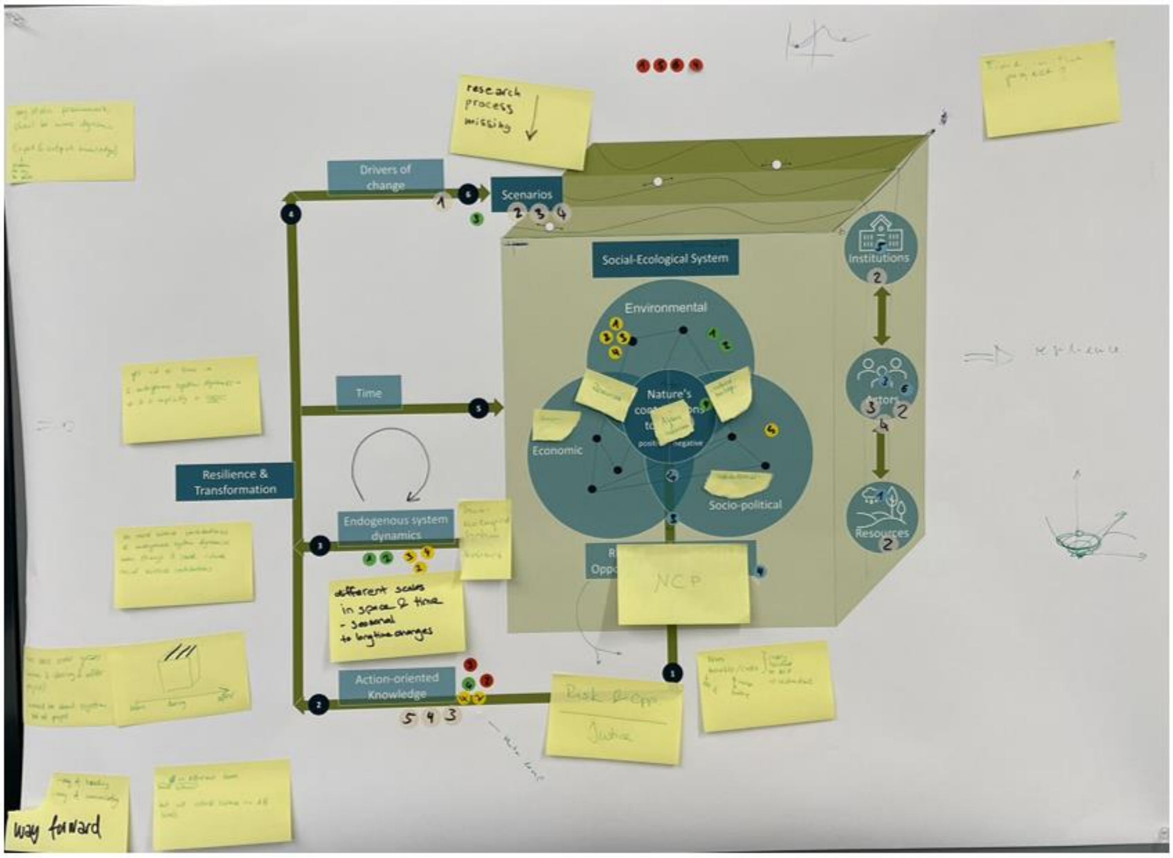

Step 9.1: CF_v

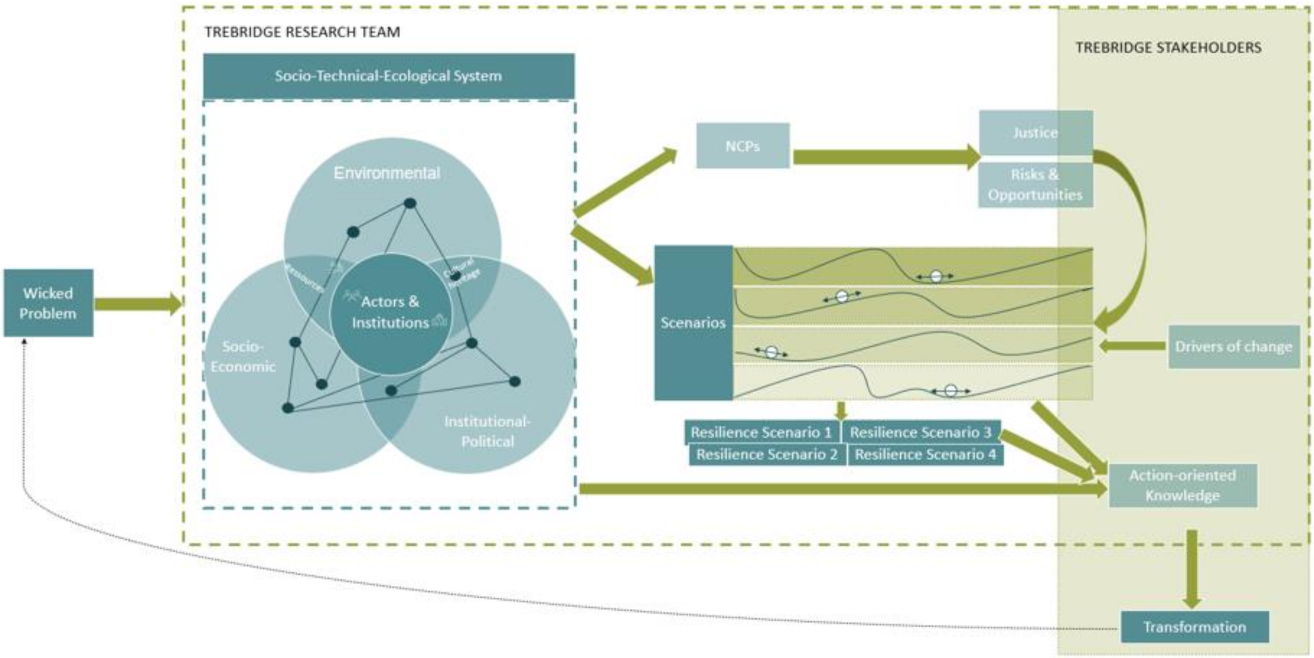

Step 9.2: CF_vi

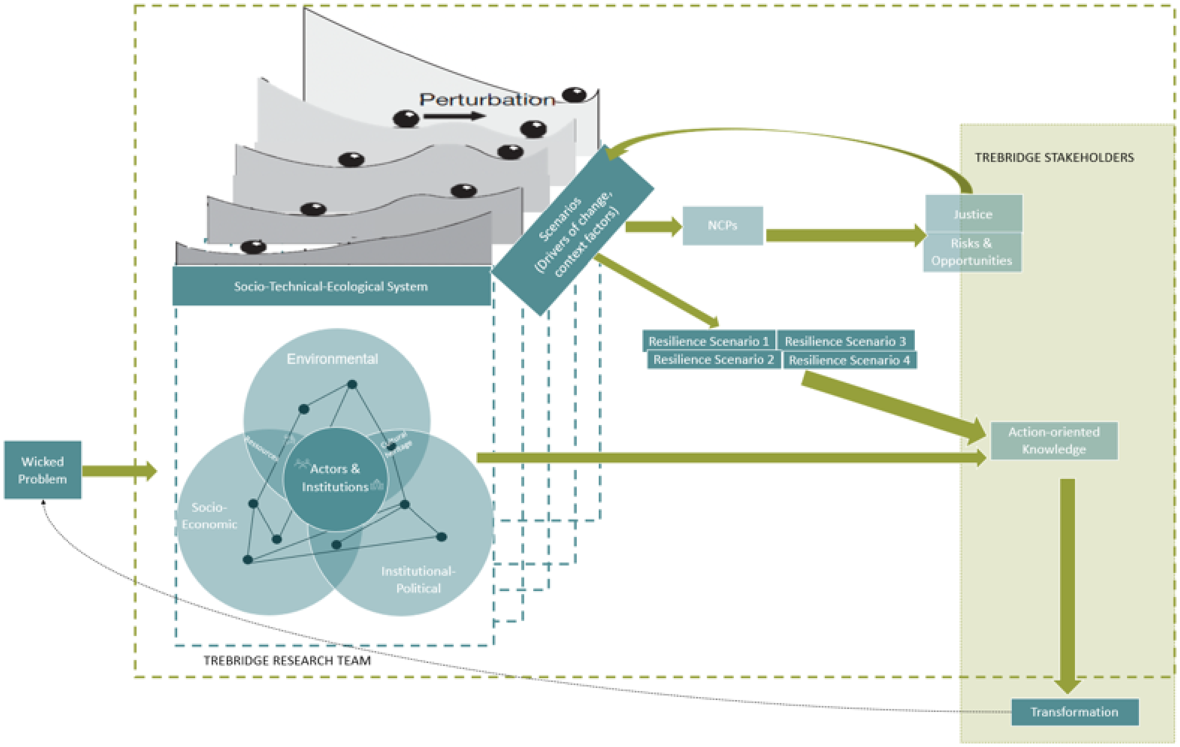

Resilience landscapes based on Scheffer et al. (2001).

Step 10.1: CF_vii

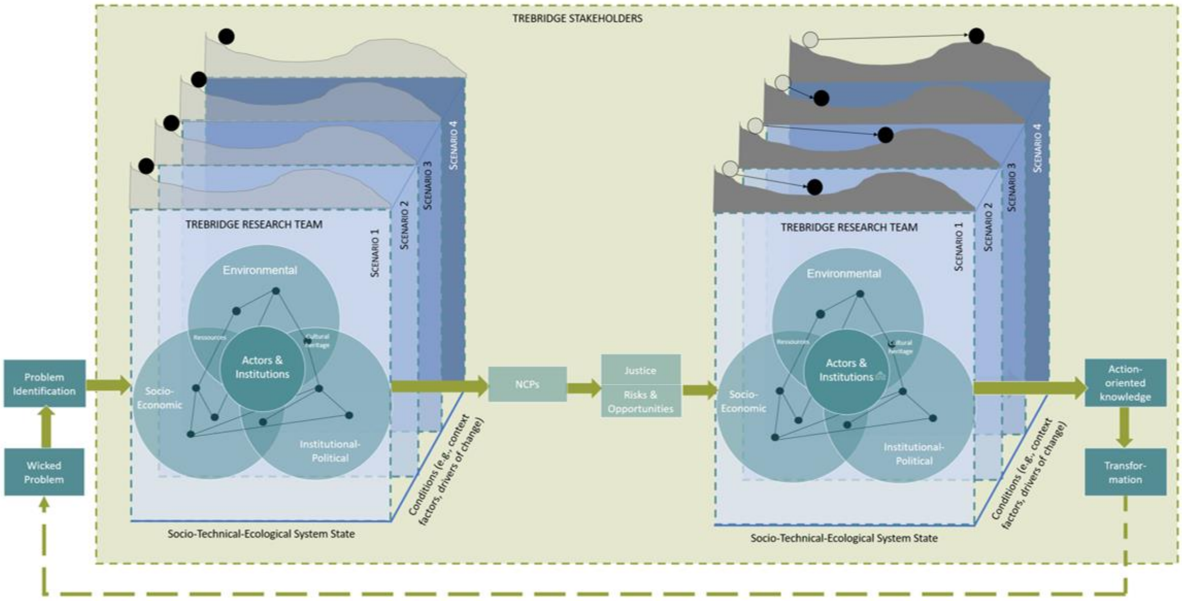

Step 10.2: CF_viii

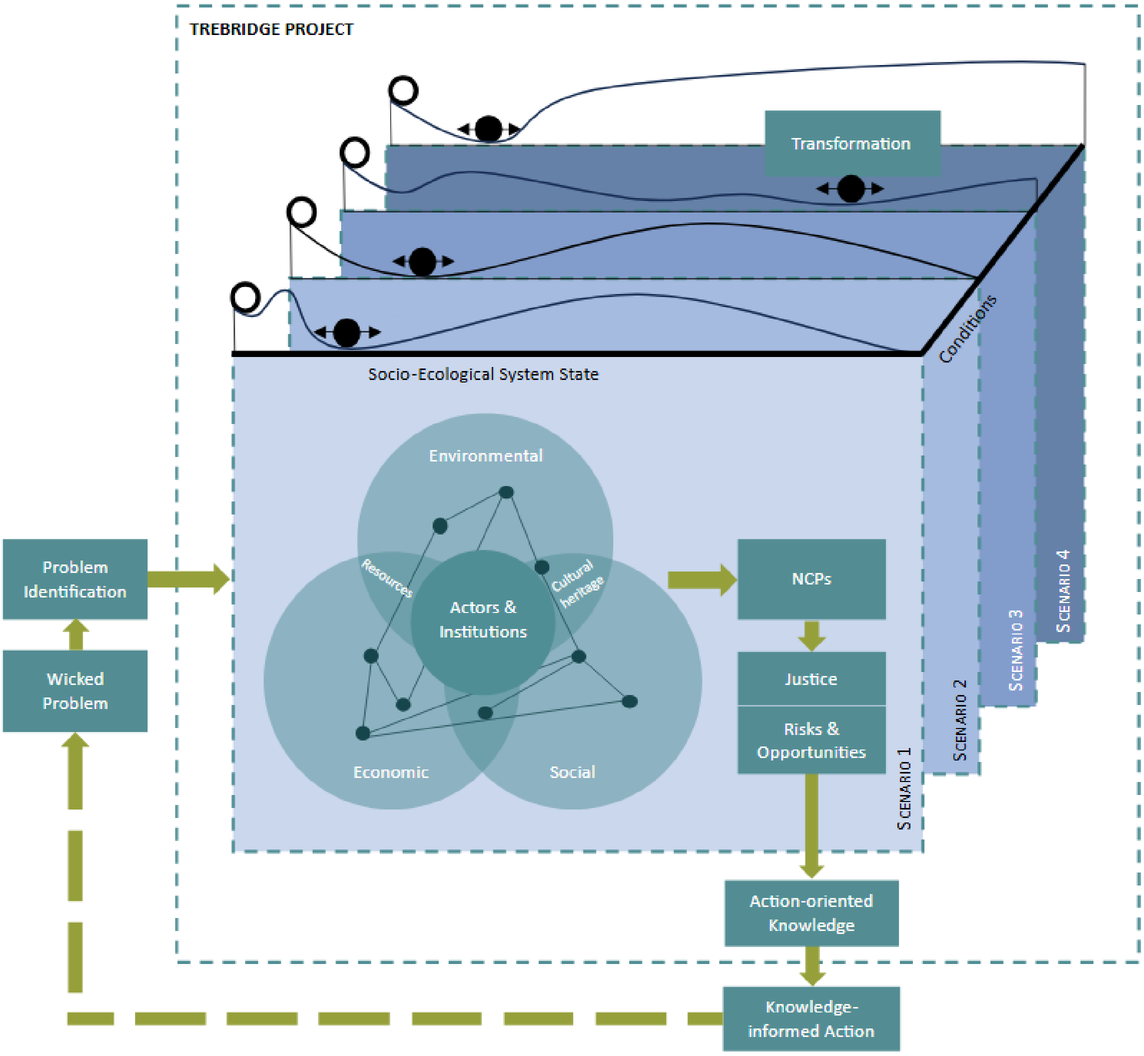

Appendix E. Survey

Thank you for taking the time to participate in our survey! This survey aims to collect feedback on the development of the conceptual system framework for the TREBRIDGE project. Your responses will provide valuable information that will help us improve our processes and contribute to a joint publication.

Completing the survey will take approximately 15 minutes. All information collected in this survey will be handled with confidentiality and used solely for the purposes stated. If you have any questions or encounter any issues during the survey, please feel free to contact us.

Introduction

A1: What is your name?

Part 1: Feedback on the Conceptual system framework

1.1 How useful do you think the conceptual system framework is as a tool to facilitate communication in the TREBRIDGE project? Please rate it below, 1 indicates not at all useful, 6 indicates very useful. Your comments are optional but appreciated.

1.2 How useful do you think the conceptual system framework is as a tool to facilitate collaborations in the TREBRIDGE project? Please rate it below, 1 indicates not at all useful, 6 indicates very useful. Your comments are optional but appreciated.

1.3 How useful do you think the conceptual system framework is as a tool to facilitate integrations from different disciplines in the TREBRIDGE project? Please rate it below, 1 indicates not at all useful, 6 indicates very useful. Your comments are optional but appreciated.

Part 2: Reflection

In this part, we are seeking to assess and enhance our approach for developing a conceptual system framework. Currently, our approach includes the following steps: 1) identify integrative terms and concepts, 2) clarify and define these integrative terms and concepts, 3) build an initial framework, 4) modify the framework through semi-structured interviews, 5) finalize the framework. We highly value your valuable insights regarding your experience and suggestions for further improving this approach.

2.1 What challenges did you encounter throughout the entire framework development process (step 1-5), and what factors may have contributed to these challenges?

2.2 What benefits and opportunities did you experience during the entire framework development process (step 1-5), and what could have caused it?

2.3 In what ways can we enhance this approach (step 1-5) to further improve the process of building the conceptual system framework?

2.4 What are you still wondering about?
